# Comparison of Heterozygosity and Genetic Diversity Between Wild and Domesticated Macadamia

**DOI:** 10.3390/biology15181627

**Published:** 2026-09-15

**Authors:** Sachini Lakmini Manatunga, Agnelo Furtado, Bruce Topp, Mobashwer Alam, Patrick J. Mason, Robert J. Henry

**Affiliations:** 1 Queensland Alliance for Agriculture & Food Innovation (QAAFI), University of Queensland, St. Lucia, QLD 4072, Australia; s.manatunga@uq.edu.au (S.L.M.); a.furtado@uq.edu.au (A.F.); p.mason1@uq.edu.au (P.J.M.); 2 ARC Centre of Excellence for Plant Success in Nature and Agriculture, University of Queensland, Carmody Rd, St. Lucia, QLD 4072, Australia; 3 Maroochy Research Facility, Queensland Alliance for Agriculture & Food Innovation (QAAFI), University of Queensland, Nambour, QLD 4560, Australia; b.topp@uq.edu.au (B.T.);; 4 VinUni Big Data Research Institute, VinUniversity, Vinhomes Ocean Park, Da Ton, Gia Lam, Hanoi 12426, Vietnam

**Keywords:** diversity, domesticated, heterozygosity, macadamia, wild

## Abstract

Macadamia is a diploid rainforest tree native to Australia. A total of 349 samples, including wild and domesticated accessions, were subjected to whole-genome sequencing, identifying 4.2 to 7.7 million, 2.4 to 7.4 million, 2.8 to 4.4 million, 1.6 to 3.2 million and 3.1 to 9.6 million single-nucleotide polymorphism (SNP) positions for *M. integrifolia*, *M. tetraphylla*, *M. ternifolia*, *M. jansenii* and the domesticated cultivars and breeding selections respectively. Overall diversity for wild *M. integrifolia*, *M. tetraphylla*, *M. ternifolia* and *M. jansenii* was 4.43%, 4.33%, 1.80% and 1.1%, respectively. Domesticated *M. integrifolia* showed an overall diversity of 3.4%, which was 1.03 percentage points lower than that of wild *M. integrifolia*. Among breeding groups, Australian breeding selections had the highest diversity, whereas Hawaiian cultivars had the lowest. Wild *M. integrifolia*, *M. tetraphylla*, *M. ternifolia* and *M. jansenii* exhibited heterozygosity ranging from 0.36% to 0.71%, 0.30% to 0.71%, 0.29% to 0.42%, and 0.19% to 0.34 respectively. Heterozygosity showed weak correlation with yield efficiency, cumulative kernel yield, and cumulative nut-in-shell yield, with a weak trend suggesting that performance may be the greatest at intermediate levels of heterozygosity. Overall, substantial genetic variation exists among the different macadamia accessions, with wild germplasm showing the greatest diversity.

## Simple Summary

Macadamia is a tree native to the rainforests of Australia and is grown around the world for its valuable nuts. Developing improved macadamia varieties requires a good understanding of the natural genetic differences found in both wild and cultivated trees. In this study, we analysed the genome-wide genetic diversity of 349 wild and cultivated macadamia trees from different breeding programmes. We found that wild macadamia trees contain much greater genetic diversity than cultivated varieties, showing that some diversity has been lost through domestication and breeding. This study provides an important resource for future research and breeding programmes.

## 1. Introduction

Macadamia is a nut crop belonging to the family Proteaceae and subfamily Grevilleoideae. The genus *Macadamia* comprises four species native to eastern Australia. Among these, *M. integrifolia* has the widest natural distribution, followed by *M. tetraphylla* and *M. ternifolia*, which occur mainly in northern New South Wales and north of Brisbane, respectively. *Macadamia jansenii* has the most restricted distribution and is confined to Bulburin National Park, north of Bundaberg [1]. Of the four species, *M. integrifolia* and *M. tetraphylla* produce edible nuts and are the primary progenitors of key commercial cultivars [2,3], whereas the remaining species are inedible due to the presence of cyanogenic glycosides [3,4]. *M. integrifolia* and *M. tetraphylla* are generally medium to large trees, whereas *M. ternifolia* and *M. jansenii* are smaller in stature. *M. integrifolia*, *M. ternifolia*, and *M. jansenii* typically have three leaves per whorl, while *M. tetraphylla* commonly has four. The species also differ in leaf margins, flower colour, and nut characteristics [1]. Nuclear phylogenetic analysis clearly distinguished the four *Macadamia* species and classified them into two main clades. *M. integrifolia* and *M. tetraphylla* grouped together in one clade, while *M. ternifolia* and *M. jansenii* formed the second clade, indicating close relationships within each pair [5]. Macadamia is partially self-incompatible, meaning that although some degree of self-compatibility exists, cross-pollination is favoured [6]. This mechanism reduces self-fertilisation and increases genetic diversity.

Recent genome assemblies provide a useful genomic context for diversity studies across the genus Macadamia. The *M. integrifolia* genome (Genome Warehouse repository GWHESFF00000000) is approximately 775 Mb and contains 47,301 predicted coding sequences (CDSs), while the *M. tetraphylla* genome (GWHESFG00000000) is approximately 795 Mb with 45,519 CDSs. The *M. ternifolia* genome (GWHESEN00000000) is approximately 780 Mb and contains 45,694 CDSs, whereas the *M. jansenii* genome (GWHESFI00000000) is approximately 773 Mb with 43,092 CDSs. Macadamia species have 14 chromosomes, and repeat analysis has shown that approximately 61% of their genomes consist of repetitive sequences [7]. These genomic resources provide an important foundation for genome-wide analyses of genetic diversity, heterozygosity, and variation among wild and domesticated macadamia populations.

The analysis of plant genetic diversity, defined as variation in genetic information within a population or species, is fundamental to the conservation of biodiversity and the study of evolution [8,9,10,11]. Recombination during sexual reproduction is one mechanism contributing to the generation and maintenance of genetic diversity and can increase genetic variation compared with asexual or vegetative propagation [11,12]. In plants, genetic diversity is also influenced by several factors such as natural selection, migration, mutation and genetic drift [13]. Populations with low genetic diversity may face a higher risk of population decline or extinction compared to a population with rich genetic diversity because it lacks the ability to adapt effectively to environmental changes such as climate change, pests, diseases, and habitat alteration [11,14,15]. Therefore, maintaining genetic diversity within a population or species can enhance its long-term survival and evolutionary adaptability [12].

The study of genetic diversity has benefitted from advances in sequencing technologies, which enable the generation of high-quality genomic data across diverse plant species [11]. These technologies facilitate the detection of millions of genome-wide DNA polymorphisms, including single-nucleotide polymorphisms (SNPs), multi-nucleotide polymorphisms (MNPs), and insertions and deletions (Indels) [16]. Among these, SNPs are the most abundant and reliable form of variations in plant genomes [17]. They occur as either “heterozygous SNPs” or “homozygous SNPs” and are valuable resources for genomic analysis and crop improvement [18].

Numerous studies have utilised SNPs derived from whole-genome sequencing to assess genetic diversity in crops such as bitter gourd [19], tobacco [17], cucumber [20], and plum [21]. In macadamia, genetic diversity has been extensively assessed using a range of molecular marker systems. Early studies used DNA fingerprinting approaches, including random amplified DNA fingerprinting (RAF) markers, to characterise genetic relationships, cultivar origins, and species composition [22,23]. High-throughput marker systems have also been applied, including DArTseq-derived silicoDArT and single-nucleotide polymorphism (SNP) markers [24]. SNP and silicoDArT markers have subsequently been used to investigate population structure, genetic diversity, and linkage disequilibrium in macadamia breeding populations [25], while genome-wide SNP markers have also been applied to characterise the genetic structure and diversity of wild germplasm and cultivated accessions [26,27,28]. However, relatively few studies have investigated genetic diversity based on SNPs identified from whole-genome sequence data. The genetic diversity of the *M. jansenii* population has been assessed using SNPs derived from whole-genome sequencing data, revealing low diversity within the species [29]. A study by Lin et al. reported diversity across 112 accessions, including wild populations, cultivars, and selected lines [30]. Furthermore, another study reported the genetic diversity of Chinese macadamia germplasm [31]. More recently, Zheng et al. investigated Chinese macadamia germplasm using fourfold degenerate SNP sites and revealed substantial genetic diversity and population differentiation [32]. However, to our knowledge, population-scale diversity in macadamia at the whole-genome level remains largely unexplored.

Heterozygosity is a key measure of genetic variation and is widely used to compare diversity within and between populations. In wild populations, it is typically reported as observed and expected heterozygosity, where expected heterozygosity is based on allele frequencies, and observed heterozygosity is derived directly from individual genotypic data [33]. Knowledge of heterozygosity across species and populations is essential for conservation biology and for enhancing agricultural productivity and long-term sustainability [34]. In general, plants with higher heterozygosity exhibit greater adaptability to biotic and abiotic stresses than those with lower heterozygosity [34]. Recently, increasing attention has been given to genome-wide heterozygosity in plants. In macadamia, isozyme marker data were initially used to determine heterozygosity in all species except *M. jansenii* [35]. Since then, numerous studies have reported heterozygosity in both wild and domesticated cultivars [22,25,26,27,31,36,37,38,39,40,41]. Molecular marker analyses have indicated high levels of heterozygosity in macadamia [40]. However, variation in heterozygosity among individual plants in wild and domesticated populations remains largely unknown.

The objective of this study was to better understand diversity within the four macadamia species and domesticated material and to compare the diversity among the individuals in wild and domesticated populations. Genome-wide SNP data were used to estimate heterozygosity and to investigate its relationship with phenotypic traits important for crop production.

## 2. Materials and Methods

### 2.1. Plant Materials

A total of 349 macadamia samples were analysed in this study. These comprised 150 wild macadamia accessions representing all four species [*M. integrifolia* (*n* = 48), *M. tetraphylla* (*n* = 56), *M. ternifolia* (*n* = 23), and *M. jansenii* (*n* = 23)] (Table S1) and 199 macadamia cultivars (Table S2) and breeding selections from the Australian macadamia breeding programme. Wild accessions were collected from an arboretum at the Maroochy Research Facility, Nambour (Queensland), and from ex situ germplasm field trials at Tiaro (Queensland) and Alstonville (New South Wales), Australia [42]. The species identity of the wild material was confirmed using previously published phylogenetic analyses based on whole-genome sequencing data [5]. Cultivars and unreleased breeding selections were collected from ex situ germplasm centres at Alstonville, Beerwah, Bundaberg, Nambour and Tiaro. Fully expanded young fresh leaves were collected in perforated bags and immediately placed under dry ice until they were stored in a −80 °C freezer at The University of Queensland, Australia.

### 2.2. DNA Extraction, Sequencing and Data Processing

Genomic DNA was extracted from leaf tissues that were initially coarsely ground and subsequently finely pulverised under cryogenic conditions using a Qiagen Tissue Lyser (MM400, Retsch GmbH, Haan, Germany). DNA extraction was performed using a modified cetyltrimethylammonium bromide (CTAB) method [43]. The quality and quantity of extracted DNA were evaluated using a Nanodrop spectrophotometer (Nanodrop Technologies, Wilmington, DE, USA) and 0.7% agarose gel electrophoresis. Whole-genome short-read sequencing was performed by BGI Hong Kong using the DNBSEQ-G400 platform (MGI Tech Co., Ltd., Shenzhen, China). A high-quality PCR-free library was prepared, and sequencing generated 150 bp paired-end reads. Raw paired-end reads were imported into CLC Genomics Workbench 23.0.05 (CLC-GWB, CLC-Bio, QIAGEN, Germantown, MD, USA). Reads from all the samples were trimmed using a quality limit of 0.01, corresponding approximately to a Phred quality score of Q20, with all other parameters set to default values. More than 98% of the trimmed reads exhibited a Phred quality score greater than 25.

### 2.3. Variant Analysis

All data analyses were performed using CLC Genomics Workbench 23.0.05 (CLC-GWB, CLC-Bio, QIAGEN). For variant analysis, species-specific reference genomes available in the Genome Warehouse (GWH) repository were selected. The *M. integrifolia* (GWHESFF00000000) reference genome was selected for all the wild (*n* = 48) and domesticated (*n* = 39) *M. integrifolia* accessions, as well as for all cultivars and unreleased breeding selections (*n* = 199). Wild *M. tetraphylla* (*n* = 56), *M. ternifolia* (*n* = 23) and *M. jansenii* (*n* = 23) were analysed using their respective reference genomes: *M. tetraphylla* (GWHESFG00000000), *M. ternifolia* (GWHESEN00000000), and *M. jansenii* (GWHESFI00000000) [7].

The trimmed paired-end reads of all the samples were individually mapped to their respective reference genomes using the “Map to reference” tool. Mapping titration, defined as the evaluation of read mapping performance across different alignment stringencies, was performed using four combinations of read length fraction (LF) and read similarity fractions (SFs) (LF 1.0 SF 0.95, LF 1.0 SF 0.9, LF 0.9 SF 0.9, LF 0.8 SF 0.8). These tests were conducted on three representative accessions from each species to determine optimal mapping parameters (Figure S1). Based on mapping efficiency, LF 1.0 and SF 0.9 were selected for all subsequent analyses.

The following parameters were applied during read mapping: masking mode “No masking”, match score “1”, mismatch cost “2”, insertion cost “3”, deletion cost “3”, non-specific match handling “map randomly”, execution mode “standard” and minimum seed length “15”. The PCR-derived duplicate reads generated during library preparation were removed from the read alignments to prevent false-positive variant calls.

Broken paired-end reads were excluded during mapping, and Indel and structural variant analysis was performed to identify potential sequencing or alignment artefacts, including insertions, deletions, inversions and tandem duplications. Local realignment was subsequently applied to improve alignment accuracy by re-mapping reads in regions surrounding insertions and deletions.

Variant calling was performed using the “Fixed ploidy variant detection” tool to identify SNPs relative to the reference genome. To determine the optimal variant calling threshold, frequency titration was conducted at five minimum allele frequency levels (40%, 35%, 30%, 25%, and 20%) (Figure S2). The minimum frequency percentage threshold represents the proportion of sequencing reads supporting a variant at a given position (calculated as count/coverage). A threshold of 25% was selected based on the stability of SNP detection across tested levels.

The following parameters were used for variant detection: required variant probability 90%, minimum coverage 10, minimum count 3, base quality filter enabled, neighbourhood radius 5, minimum central quality 20, minimum neighbourhood quality 15, and direction frequency 5%.

Following variant detection, multi-nucleotide variants (MNVs), insertions, deletions, and replacements (complex sequence changes in which a reference sequence segment is replaced by an alternative sequence) were identified. SNPs were further classified as heterozygous when the alternative allele was supported by 25–75% of the sequence reads at that position or homozygous when the allele frequency was 100% with >20× coverage. These variants were filtered separately for the whole-genome and coding sequence (CDS) regions across all samples.

### 2.4. Genetic Diversity Analysis

Diversity analysis was conducted separately for 16 different populations, including wild *M. integrifolia* (*n* = 48), wild *M. tetraphylla* (*n* = 56), *M. ternifolia* (*n* = 23), *M. jansenii* (*n* = 23), domesticated *M. integrifolia* (*n* = 39), and several domesticated groups: cultivars from the Hawaiian Agricultural Experiment Station (HAES; *n* = 37), those from Hidden Valley Plantation (HVP; *n* = 12), heritage cultivars (*n* = 17), cultivars bred by Norm Greber (*n* = 9), cultivars bred by Backer (*n* = 4), cultivars bred by Malawi (*n* = 2), and cultivars bred by Ian McConachie (*n* = 7). Additional groups included Australian national breeding programme accessions (NMBPAs; *n* = 108), a whole domesticated gene pool (*n* = 199) and cultivars and unreleased breeding selections grouped in clade I (*n* = 4) and clade II (*n* = 195) based on nuclear phylogenetic analysis from our previous publication [44]. Clade I comprised accessions clustering separately from the main domesticated group, while clade II contained the majority of domesticated cultivars and breeding selections.

All analyses were performed using CLC Genomics Workbench 23.0.05 (CLC-GWB, CLC-Bio, QIAGEN, Germantown, MD, USA). Genetic diversity was assessed using heterozygous SNPs, defined as sites where the alternative allele was supported by 25–75% of sequence reads, and homozygous SNPs, defined as sites with 100% allele frequency and >20× coverage.

For each accession within a given population, heterozygous and homozygous SNPs were merged into a single dataset. These accession-level datasets were then combined at the population level using a frequency threshold of 0% (Figure S3 step 01), ensuring that all variant positions across accessions were retained. To identify polymorphic (diverse) positions, non-diverse SNPs were first removed. Non-diverse homozygous SNPs (positions identical across all accessions) were filtered based on sample count equal to the total number of samples and zygosity classified as “homozygous” (Figure S3 step 02). Similarly, non-diverse heterozygous SNPs were identified and removed using equivalent criteria (Figure S3 step 03). These non-diverse SNPs were combined (Figure S3 step 04) and excluded from further analysis, leaving only polymorphic SNPs (Figure S3 step 06).

Polymorphic SNPs were further categorised into two groups. “High-coverage SNPs” were defined as positions where accessions exhibited a mixture of heterozygous and homozygous variants or where some accessions carried variant alleles while others retained the reference allele (Figure S3 step 05). The remaining polymorphic SNPs were classified as “low-coverage SNPs” (Figure S3 step 07).

To reduce potential artefacts, low-coverage SNPs were subjected to additional filtering. Homozygous (Figure S3 step 08) and heterozygous (Figure S3 step 10) positions were processed separately, and potential noise arising from unmapped reads or variant calling errors was identified using a mutation analysis pipeline [45] (Figure S3 step 11). Heterozygous SNP positions that matched the reference across all the accessions were combined (Figure S3 step 13) and filtered against the total set of diverse SNPs using the option “Keep variants with exact match found in the track of known variants” (Figure S3 step 15). Similarly, homozygous SNP positions matching the reference were combined (Figure S3 step 12) and filtered against the total set of diverse SNPs using the same option (Figure S3 step 14).

The total number of diverse heterozygous SNP positions was calculated by combining the number of heterozygous SNP positions matching the reference across all accessions and high-coverage SNPs (Figure S3 step 16). The total number of diverse SNP positions was calculated by combining validated heterozygous SNPs, homozygous SNPs, and high-coverage SNPs (Figure S3 step 17). Diversity was then calculated as the percentage of polymorphic loci relative to the total genomic size. Individual diversity values were calculated by extracting accession-specific variant data using the option “Origin track with the name of the accession”. Accession-specific unique polymorphic SNPs were identified by selecting variants with a sample count ≤ 1, separately for heterozygous and homozygous SNPs. Diversity between wild and domesticated *M. integrifolia* was compared using an independent two-sample *t*-test. Statistical analyses were performed in Microsoft Excel using the Data Analysis ToolPak.

### 2.5. Heterozygosity

Heterozygosity for each sample was calculated as a percentage of heterozygous single-nucleotide variant (SNV) positions relative to the total number of positions in the reference genome. Heterozygous SNV positions were identified by filtering using “Reference allele contains No”.

### 2.6. Phenotypic Data Collection Standardisation and Correlation Analysis

Historical phenotypic datasets collected in Queensland, Australia, were utilised in this study. Data were obtained for six traits (cumulative nut-in-shell (NIS) yield at age 8, cumulative kernel yield at age 8, kernel recovery (kernel weight/nut-in-shell weight), canopy volume at age 6, tree height at age 6 and yield efficiency (kernel yield /tree volume)). These data were available for 79 accessions (Table S3), comprising both parental lines and the second-generation progeny from the Australian macadamia breeding programme, evaluated across 14 trials at nine locations [46]. Phenotypic data were standardised to account for location effects and analysed using ASReml-R v4.0, following the methodology described in Topp et al., 2014 [47]. Pearson’s correlation coefficients were calculated to assess the relationships between heterozygosity and the six phenotypic traits.

## 3. Results

### 3.1. Sequencing and Mapping of Reads to Reference Genome

The sequencing of 349 macadamia accessions generated between 98,509,980 and 260,592,896 raw reads per sample, with sequence coverage ranging between 18.23× and 50.11× (Table S4). After quality trimming at a threshold of 0.01, the number of reads per sample ranged from 93,695,956 to 247,617,841.

Read mapping was performed using four species-specific reference genomes. For wild *M. integrifolia* populations (*n* = 48), as well as wild trees from mixed/hybrid populations (*n* = 4), wild macadamia trees of uncertain species identity (*n* = 9), a wild hybrid (*n* = 1), a wild admixture (*n* = 1), and all cultivars and breeding selections (*n* = 199), the percentage of trimmed paired-end reads successfully mapped to the *M. integrifolia* genome ranged from 70.2% to 93.9% (Table S5). The lowest mapping percentage was observed for Mac_318, which is known to be a wild macadamia tree of uncertain species identity. For the wild *M. tetraphylla* population (*n* = 56), the mapping percentage to the *M. tetraphylla* reference genome ranged from 80.8% to 93.4% (Table S6). Similarly, for *M. ternifolia* (*n* = 23), mapping percentages ranged from 76.5% to 95.1% (Table S7). For the *M. jansenii* population (*n* = 23), the mapping percentages ranged from 87.3% to 95.4% (Table S8).

### 3.2. Detection of DNA Polymorphisms

Among the wild *M. integrifolia* population, 4,180,786 to 7,661,269 SNP positions were identified across 48 accessions, corresponding to 5.39–9.88 SNP per kb of the *M. integrifolia* genome (Figure S4) (Table 1). The number of MNPs, insertions, deletions and replacements ranged from 221,995 to 326,706; 355,810 to 472,711; 366,919 to 536,547 and 31,772 to 43,398, respectively (Table S9). The highest total number of SNPs was observed in Mac_035, whereas the lowest was observed in Mac_171. Mac_091 exhibited the highest number of heterozygous SNPs, while Mac_035 had the highest number of homozygous SNPs (Table S10). The lowest numbers of heterozygous and homozygous SNPs were observed in Mac_250 and Mac_130, respectively. At the CDS level, SNP positions ranged from 159,772 to 273,486 (Table S9).

For the wild *M. tetraphylla* population, between 2.9 million and 8.8 million variant positions were identified across 56 accessions (Table 1). At the whole-genome level, SNPs (2,446,762–7,356,187) (3.07 SNP/kb to 9.25 SNP/kb) represented the predominant variant type, followed by deletions (203,140–574,046), insertions (169,567–477,646), MNPs (110,618–395,403) and replacements (12,586–48,490) (Figure S5, Table S11). The highest number of SNPs (12,550,003) was observed in Mac_236, while the lowest was recorded in MacP_15 (4,832,434) (Table S12). Heterozygous SNPs ranged from 4,742,286 to 11,333,633, with the highest value observed in Mac_236. Homozygous SNPs ranged from 90,148 to 2,777,121, with the highest number recorded in Mac_030, which is believed to be a planted tree from wild trees. At the CDS level, SNP positions ranged from 83,793 to 277,573 (Table S11).

Within the *M. ternifolia* population (*n* = 23), between 3.6 million and 5.2 million variant positions were identified at the whole-genome level, corresponding to 3.62–5.62 SNP positions per kb of the *M. ternifolia* genome (Figure S6) (Table 1). The number of MNPs, insertions, deletions and replacements ranged from 88,709 to 170,484; 173,145 to 335,609; 200,108 to 375,617; and 12,833 to 26,513, respectively (Table S13). Mac_300 exhibited the highest number of heterozygous SNPs (6,556,435), while Mac_126 showed the lowest (4,649,136) (Table S14). Mac_332 had the highest number of homozygous SNPs (1,411,730), whereas MacP_12 had the lowest (11). At the CDS level, between 1.1 M and 1.8 M variant positions were identified, with SNPs representing the predominant variant type (106,034–166,120) (Table S13).

Among the 23 *M. jansenii* accessions, the total number of variant positions ranged from 1.9 million to 3.8 million (Table 1). SNP density ranged from 2.07 to 4.12 per kb (Figure S7). MNPs ranged from 66,284 to 119,204, insertions from 121,641 to 241,370, deletions from 144,258 to 281,908, and replacements from 8330 to 18,418 (Table S15). The highest number of SNPs was observed in Mac_282, while the lowest was observed in MacP_16. Mac_282 exhibited the highest number of heterozygous SNPs, whereas MacP_02 showed the highest number of homozygous SNPs (Table S16). At the CDS level, SNP counts ranged from 59, 297 to 111,702 (Table S15).

A variant analysis of domesticated macadamia accessions (*n* = 199) revealed a wide range of polymorphism, with total variant counts ranging from 3.8 million to 11.3 million. SNP density ranged from 4.02 to 12.4 SNPs per kb of the *M. integrifolia* genome (Figure S8) (Table 1). MNPs ranged from 97,087 to 476,580, insertions from 239,499 to 537,367, deletions from 274,691 to 613,841 and replacements from 18,198 to 55,693 (Table S17). Mac_333 from the Australian National Macadamia Breeding Program Accession (NMBPA), identified as a wild–domestic hybrid, exhibited the highest number of SNPs and heterozygous SNPs (Table S18). The cultivar “Minimacca” (Mac_347) showed the highest number of homozygous SNPs, whereas “HAES 741” (Mac_135) exhibited the lowest. At the CDS level, SNV counts ranged from 107,053 to 366,556 (Table S17).

### 3.3. Genetic Diversity Analysis and Comparison

Genetic diversity was assessed as the percentage of polymorphic loci relative to the reference genome across different population groups. Diversity for the wild *M. integrifolia* population is presented in Table S19. Total genetic diversity within this population was 4.43%, with individual values ranging from 0.53% to 0.97% (Figure S9) (Table 2). The lowest diversity was observed in Mac_163, while the highest was observed for Mac_035. The highest number of accession-specific, unique polymorphic heterozygous SNP positions (733,189) was recorded in Mac_344 (Table S20), whereas the highest number of accession-specific, unique polymorphic homozygous SNP positions (34,686) was observed in Mac_250.

For the domesticated *M. integrifolia* population, diversity ranged from 0.39% to 0.96%, with a total diversity of 3.40% across 39 accessions (Table S21; Figure S10). The lowest and the second lowest were recorded for the cultivar HAES 741 (Mac_135 and Mac_195, respectively) with respect to its HAES 741 reference genome (*M. integrifolia*). The highest diversity was observed in the cultivar “Daddow” (Mac_072). Accession-specific, unique polymorphic heterozygous SNP positions ranged from 15,303 (Mac_055) to 1,497,179 (Mac_156) (Table S22), with the highest and lowest values observed in HAES 800 (Mac_156) and HAES 807 (Mac_055), respectively. The highest number of accession-specific, unique polymorphic homozygous SNP positions was recorded in cultivar D1 (Mac_087).

A comparison between wild and domesticated *M. integrifolia* indicated greater diversity in wild germplasm. An independent two-sample *t*-test confirmed a statistically significant difference between the two groups (*p* = 9.81367 × 10^−7^, Cohen’s d = 1.17) (Figure S11).

Wild *M. tetraphylla* populations exhibited a total genetic diversity of 4.33% across 56 accessions, with individual values ranging from 0.31% to 0.92% (Table S23; Figure S12). The lowest diversity was observed in MacP_015, while the highest was observed in Mac_030. Accession-specific, unique polymorphic heterozygous SNP positions ranged from 25,308 to 2,863,676 (Table S24), and homozygous SNP positions ranged from 123 to 614,911.

The *M. ternifolia* population showed diversity values ranging from 0.36% to 0.55%, with a total diversity of 1.80% across 23 accessions (Figure S13; Table S25). The lowest diversity was observed in two accessions, MacP_12 (reference genome accession) and Mac_126, and the highest diversity was observed in Mac_300 and Mac_310. Accession-specific, unique polymorphic heterozygous and homozygous SNP positions ranged from 27,588 to 419,584 and 0 to 15,430, respectively (Table S26).

For the isolated *M. jansenii* population, total genetic diversity was 1.1%, with individual values ranging from 0.20% to 0.40% (Table S27; Figure S14). The highest number of accession-specific, unique polymorphic heterozygous SNP positions (94,407) was observed in MacP_04 (Table S28), while the highest number of homozygous SNP positions (2103) was observed in Mac_218.

Across domesticated macadamia accessions, total genetic diversity was 6.10% for 199 accessions (Table S29). Individual diversity values ranged from 0.4% to 1.24% (Figure S15). The highest diversity was observed in Mac_348, a wild–domesticated hybrid, whereas the lowest was observed in Mac_135. Accession-specific, unique polymorphic heterozygous SNP positions ranged from 3177 to 434,165 (Table S30), while the homozygous SNP positions ranged from 0 to 29,551. Notably, 19 accessions (Mac_005, Mac_016, Mac_093, Mac_102, Mac_121, Mac_133, Mac_135, Mac_137, Mac_160, Mac_162, Mac_166, Mac_176, Mac_186, Mac_190, Mac_195, Mac_203, Mac_269, Mac_285 and Mac_322) showed no accession-specific unique polymorphic homozygous SNP positions relative to the reference HAES 741 *M. integrifolia* reference genome.

A subgroup analysis of domesticated germplasm revealed variation among breeding programmes (Table 3). Hawaiian cultivars (*n* = 37) exhibited diversity values ranging from 0.39% to 0.96%, with a total genetic diversity of 3.33% (Table S31; Figure S16). The highest diversity was observed in cultivar HAES 853, whereas the lowest number of accession-specific, unique polymorphic SNP positions (both heterozygous and homozygous) was recorded in HAES 849 (15,673 SNPs) (Table S32). HVP cultivars (*n* = 12) showed a total genetic diversity of 2.31% (Table S33), with values ranging from 0.56% (Mac_295; cultivar A9/9) to 1.02% (Mac_061; cultivar A16) (Figure S17). Accession-specific, unique SNPs are presented in Table S34. Heritage cultivars (*n* = 17) ranged from 0.52% to 1.05%, with the highest diversity observed in Mac_230 (Table S35; Figure S18). The cultivar “Beaumont” exhibited the highest number of accession-specific unique SNPs (Table S36). Total genetic diversity for cultivars bred by Norm Greber (*n* = 9), Backer (*n* = 4), Malawi (*n* = 2), and Ian McConachie (*n* = 7) was 2.53% (Table S37; Figure S19), 0.96% (Table S38; Figure S20), 0.46% (Table S39; Figure S21) and 2.03% (Table S40; Figure S22) respectively. The highest number of accession-specific, unique polymorphic SNP positions for cultivars bred by Norm Greber, Backer, Malawi, and Ian McConachie was observed in Mac_187 (1,709,907) (Table S41), Mac_170 (1,979,851) (Table S42), Mac_158 (1,866,075) (Table S43) and Mac_070 (2,233,011) (Table S44).

Australian breeding selections (NMBPA) exhibited 5.16% diversity across 108 accessions, with values ranging from 0.43% to 1.23% (Table S45; Figure S23). The highest number of accession-specific, unique polymorphic SNP positions was observed in Mac_348 (Table S46). Comparative analysis across domesticated groups indicated that NMBPAs had the highest diversity, whereas HAES cultivars showed the lowest (Table S29; Figure S24). Other groups, such as HVP cultivars, heritage cultivars, cultivars bred by Norm Greber, cultivars bred by Backer and cultivars bred by Ian McConachie, showed moderate to high diversity.

Moreover, cultivars and breeding selections grouped in clade I (*n* = 4) in nuclear phylogenetic analysis from our previous publication [44] exhibited a total genetic diversity of 1.13%, with values ranging from 0.46% to 0.63% (Table S47; Figure S25). Clade II accessions (*n* = 195) showed diversity ranging from 0.4% to 1.13% (Table S49; Figure S26). The highest number of accession-specific SNP positions in clade II was observed in “Young1” (Mac_081) (Table S50). A comparison between clades is presented in Figure S27.

### 3.4. Heterozygosity Calculation

**Figure 1:**
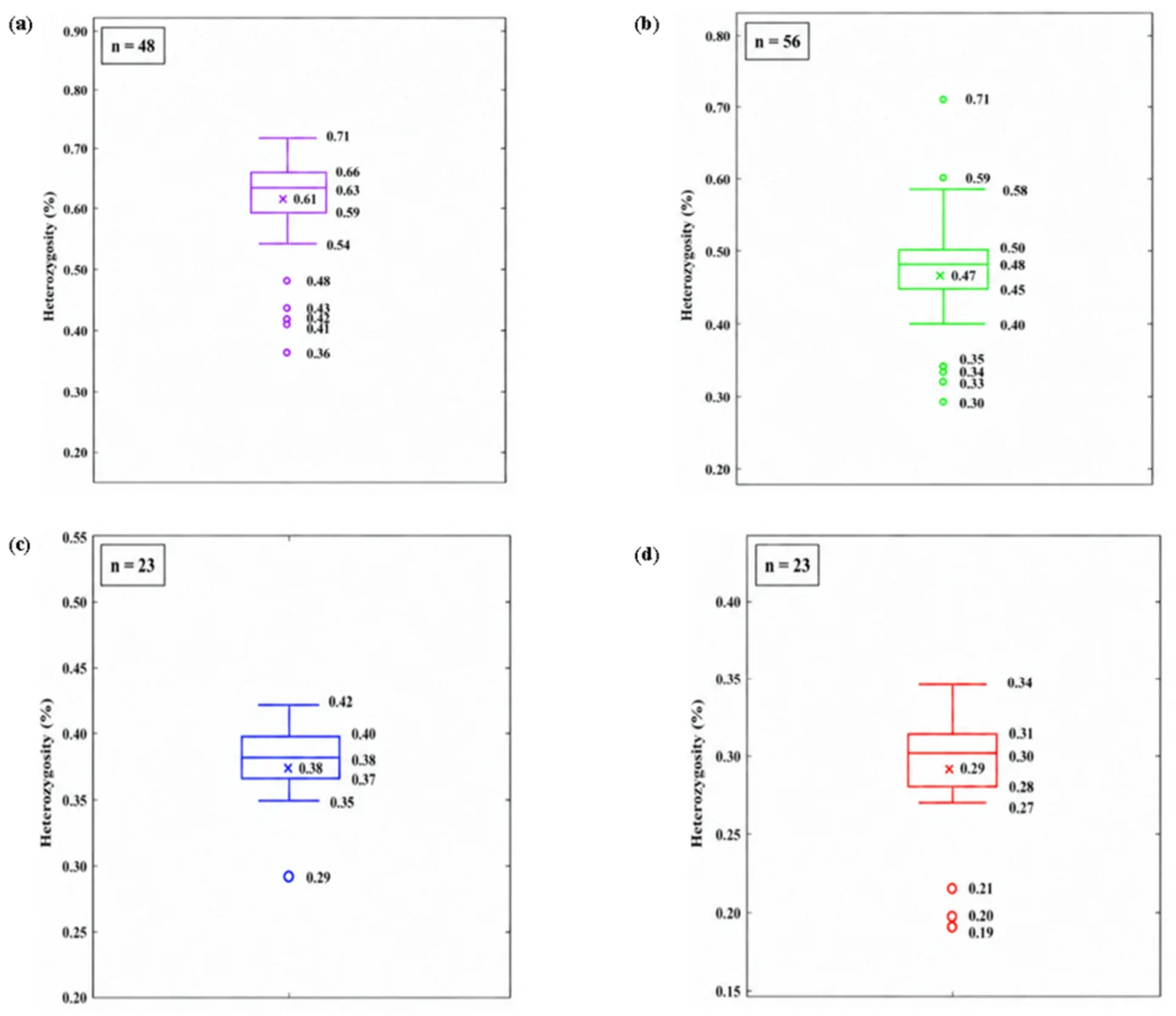
Distribution of heterozygosity. (**a**) Heterozygosity across wild *M. integrifolia* (**b**) Heterozygosity across wild *M. tetraphylla*. (**c**) Heterozygosity across wild *M. ternifolia*. (**d**) Heterozygosity across wild *M. jansenii*.

In the wild *M. integrifolia* population, heterozygosity (expressed as the percentage of heterozygous SNP positions relative to the reference genome) reached a maximum of 0.71%, observed in Mac_052 and Mac_091 (Table S51). Most accessions exhibited values between 0.54% and 0.71%, with a small number of lower-value outliers (Mac_250, Mac_171, Mac_058, Mac_163, Mac_139) (Figure 1a). For the wild *M. tetraphylla* population, the highest heterozygosity value was observed in Mac_236, while the lowest values were recorded in Mac_108 and MacP_15 (Table S52). Most accessions ranged between 0.40% and 0.58%, with several lower-value outliers (MacP_15, Mac_108, Mac_199, MacP_14, Mac_115, Mac_117, Mac_315) and a few higher-value outliers (Mac_238, Mac_236) (Figure 1b). In the *M. ternifolia* population, most accessions exhibited heterozygosity values between 0.35% and 0.42%, with Mac_126 representing a lower-value outlier (Figure 1c). The highest value (0.42%) was observed in Mac_300 (Table S53). The *M. jansenii* population showed relatively low heterozygosity, ranging from 0.19% to 0.34%. Three accessions (Mac_216, Mac_217 and MacP_16) exhibited lower values and were identified as outliers (Figure 1d; Table S54). Domesticated macadamia accessions exhibited a broader range of heterozygosity, with most values between 0.33% and 0.95%. A small number of accessions (Mac_350, Mac_348, Mac_333) showed comparatively higher values (Figure S28; Table S55).

### 3.5. Correlation Between Heterozygosity and Phenotypic Traits

**Figure 2:**
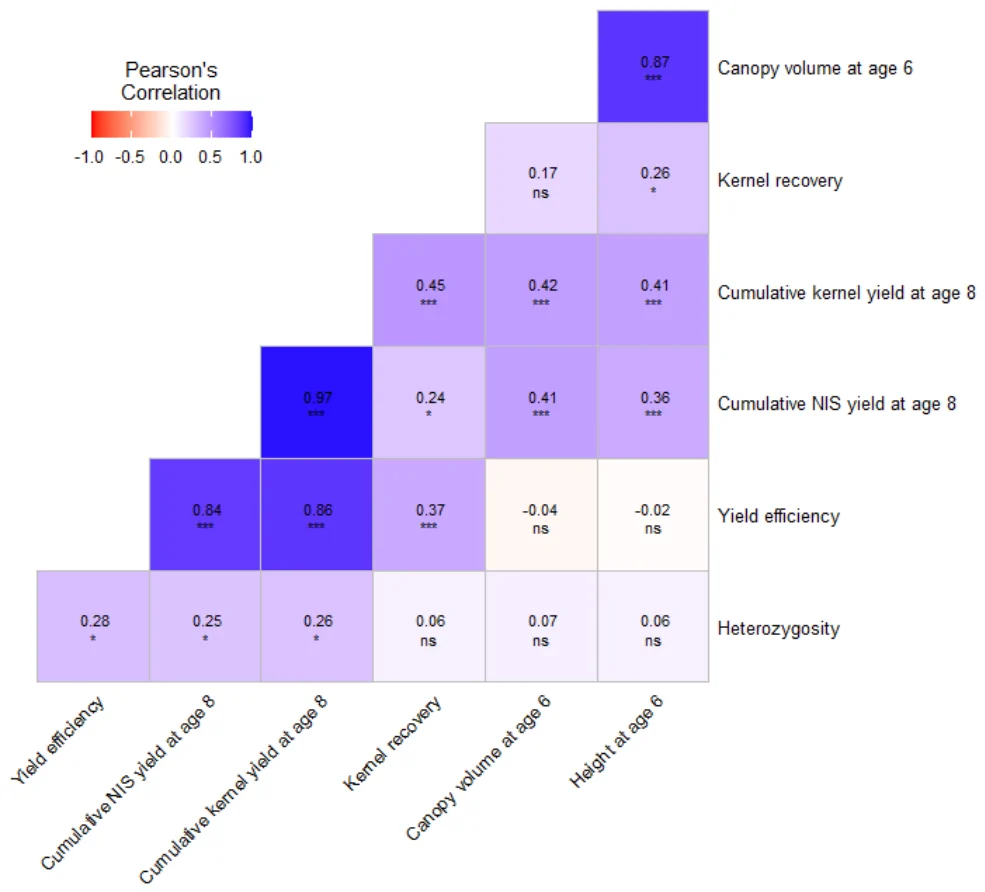
Correlation analysis results. ns: *p* ≥ 0.05, * *p* < 0.05 and *** *p* < 0.001.

Significant correlations were observed for yield efficiency (kernel yield/tree volume), cumulative kernel yield at age 8, and cumulative NIS yield at age 8 (Figure 2). Yield efficiency (kernel yield/tree volume) showed the strongest association with heterozygosity (r = 0.28, *p* < 0.05). Cumulative kernel yield at age 8 (r = 0.26, *p* < 0.05) and cumulative NIS yield at age 8 (r = 0.25, *p* < 0.05) also exhibited statistically significant correlations. No significant relationships were observed between heterozygosity and tree height at age 6, canopy volume at age 6 or kernel recovery percentage.

**Figure 3:**
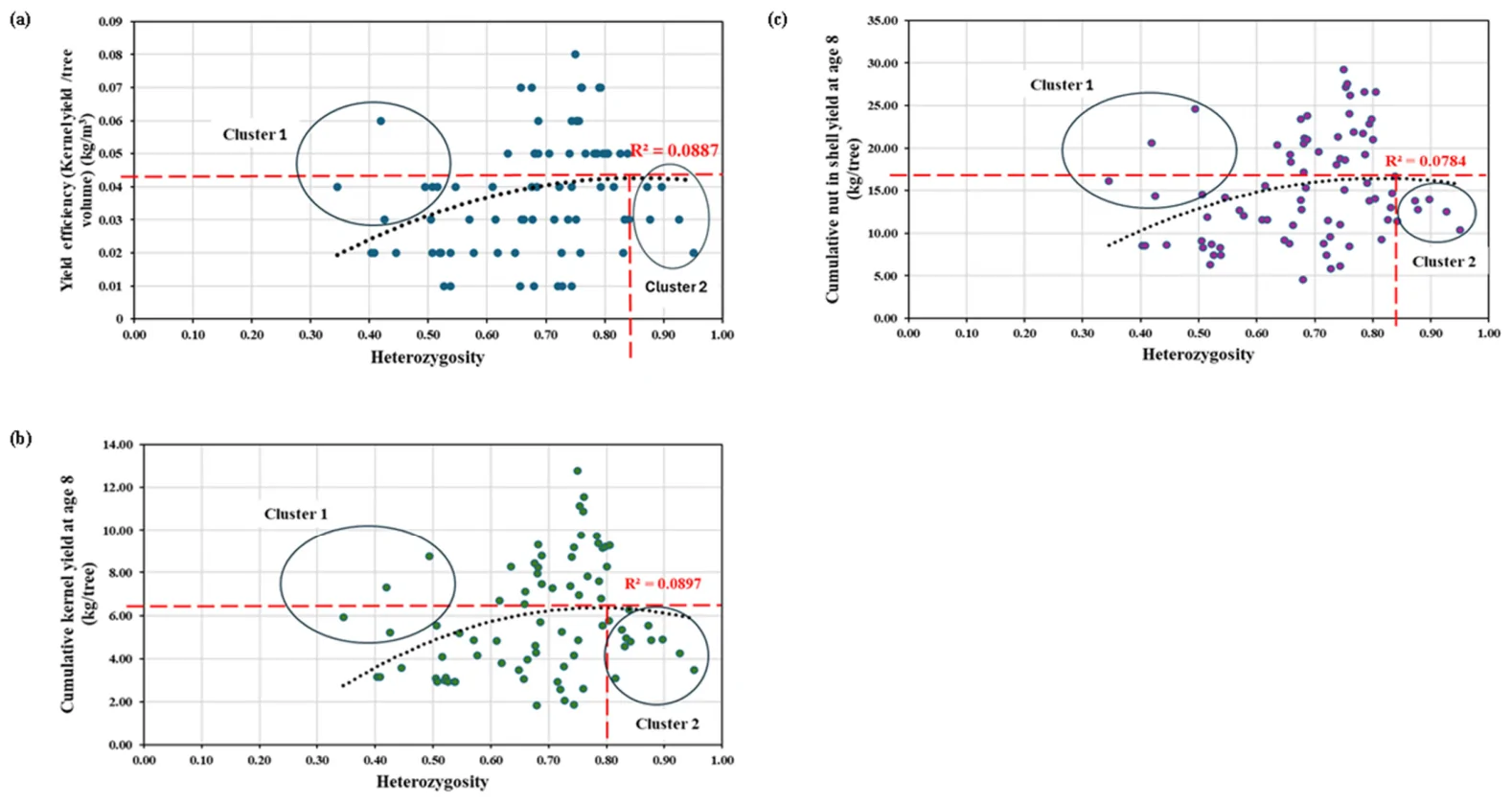
Relationship between heterozygosity and phenotypic traits. (**a**) Relationship between heterozygosity and yield efficiency (kernel yield/tree volume). (**b**) Relationship between heterozygosity and cumulative kernel yield at age 8. (**c**) Relationship between heterozygosity and cumulative nut-in-shell (NIS) yield at age 8. X-axis represents heterozygosity (%) in all three graphs. Y-axis represents (**a**) yield efficiency (kg/m^3^), (**b**) cumulative kernel yield (kg/tree), and (**c**) cumulative nut-in-shell yield (kg/tree).

The relationship between heterozygosity and yield efficiency (Figure 3a), cumulative kernel yield at age 8 (Figure 3b) and cumulative NIS yield at age 8 (Figure 3c) exhibited a nonlinear trend. In each case, performance appeared to increase with heterozygosity up to a certain level, followed by a decline. The dotted curve line in Figure 3a–c indicates this trend, with a low coefficient of determination (R^2^ = 0.0887, 0.0897, and 0.0784), suggesting a weak but discernible curvilinear relationship.

Two general patterns were observed. Some accessions exhibited relatively low heterozygosity with higher performance, while others showed higher heterozygosity with lower performance. Cluster 1 in Figure 3a–c represents accessions with low heterozygosity and high performance, whereas cluster 2 represents accessions with high heterozygosity and low performance. In Figure 3a, cluster 1 includes Mac_132, Mac_144, Mac_159, Mac_161, Mac_164, Mac_194 and Mac_198, exhibiting low heterozygosity and high yield efficiency, whereas cluster 2 comprises Mac_082, Mac_186, Mac_230, Mac_280 and Mac_294, showing high heterozygosity and low yield efficiency. In Figure 3b, a similar set of accessions to those in cluster 1 in Figure 3a, except for Mac_144 and Mac_161, exhibits low heterozygosity and high cumulative kernel yield. In contrast, cluster 2 consists of Mac_010, Mac_048, Mac_088, Mac_096, and Mac_176 together with the accessions identified in cluster 2 in Figure 3a and shows high heterozygosity and low cumulative kernel yield. In Figure 3c, low heterozygosity and high cumulative NIS yield were observed for five accessions (Mac_132, Mac_159, Mac_164, Mac_194 and Mac_198) (Figure 3c), whereas high heterozygosity and low cumulative NIS yield were observed for Mac_082, Mac_186, Mac_230, Mac_280 and Mac_294.

To assess whether these patterns were influenced by sequencing depth or mapping quality, mapping percentages were compared between accessions with contrasting heterozygosity levels. No substantial differences were observed (Figure S29), indicating that the observed trends were unlikely to be driven by technical bias. For traits that were not significantly associated with heterozygosity (tree height at age 6, canopy volume at age 6 and percentage of kernel recovery), only weak linear trends were observed (Figure S30).

## 4. Discussion

The evaluation of genetic diversity is widely used to characterise wild germplasm and breeding materials and to support conservation management and breeding programmes [48,49]. Several approaches are available to quantify diversity, including the percentage of polymorphic loci, polymorphism information content (PIC), the mean number of alleles per locus and total gene diversity [13]. In the present study, diversity was evaluated at both the individual and population levels based on the proportion of loci exhibiting allelic variation.

Diversity was identified among domesticated accessions from several breeding programmes. The highest diversity was exhibited in Australian breeding selections (NMBPA) compared with HVP cultivars, heritage cultivars, cultivars bred by Norm Greber, cultivars bred by Backer, cultivars bred by Ian McConachie, cultivars bred by Malawi and HAES cultivars. These findings align with previous research based on whole-genome SNP data, which reported the highest diversity in Australian accessions (average number of nucleotide differences per site: Pi = 1.818 × 10^−3^) and the lowest diversity in Hawaiian accessions (Pi = 1.496 × 10^−3^) [31]. Additionally, a study using SSR markers found that Australian HVP and pre-commercial selections had the second highest diversity (number of alleles per locus: Na = 8.46) following South Africa’s commercial and non-commercial local selections (Na = 9.31) [27]. Collectively, these results highlight the broad genetic base of Australian breeding materials, likely reflecting their composite origin and continued introgression from diverse germplasm sources, and provide a valuable foundation for future genetic improvement and resilience in macadamia.

This study also examined diversity at the population and individual accession levels within the four wild populations. Previous marker-based studies have reported that *M. tetraphylla* exhibited the highest diversity (Na = 15.73), followed by *M. integrifolia* (Na = 13.82), *M. ternifolia* (Na = 10.73) and *M. jansenii* (Na = 2.23) [26]. Another study also reported that *M. integrifolia* and *M. tetraphylla* exhibited higher diversity compared to *M. ternifolia* and *M. jansenii* [28]. The present study extends these findings by providing a finer-scale assessment of diversity at the individual accession level across natural populations.

A previous study of northern, central and southern Australian populations of *M. integrifolia* estimated genetic diversity using the average number of nucleotide differences per site. The central population (*n* = 15) showed the highest value (4.05 × 10^−4^), followed by the southern population (*n* = 10; 2.87 × 10^−4^) and the northern population (*n* = 10; 2.76 × 10^−4^) [30]. SNPs in that study were identified using the Genome Analysis Toolkit (GATK) pipeline relative to the cultivar “Kau” (HAES 344) reference genome. In contrast, the present study estimated genetic diversity at the individual level using the percentage of polymorphic loci and identified Mac_035 as having the highest diversity. The observed differences may be due to differences in the diversity metrics, level of analysis, and variant calling approaches used.

This study suggests that Mac_030, which is known as a planted tree, exhibits higher diversity compared with the *M. tetraphylla* genome. The previous study identified Mac_030 as *M. tetraphylla* (corresponding to M259 in the previous study) [28]. However, our results show that Mac_030 has a comparatively higher number of accession-specific unique polymorphic SNP positions (including both heterozygous and homozygous). Therefore, additional analysis is required to confirm its status as a pure *M. tetraphylla* accession.

Within the *M. ternifolia* population, two representatives of the reference accession were analysed (Mac_309 and MacP_12). As expected, MacP_12, the reference genome accession, exhibited the lowest diversity and no homozygous polymorphic positions.

For *M. jansenii*, the diversity estimates obtained in this study differ slightly from those reported previously [29]. We assessed diversity in the same eight accessions. Among these, six (MacP_01, MacP_02, MacP_4, MacP_05, MacP_06 and MacP_07) exhibited slightly higher diversity than previously reported, whereas the remaining two accessions (MacP_03 and MacP_16) showed lower diversity values. The overall diversity in the present study (1.10%) was higher than that reported previously (0.92%), possibly due to the larger population size [28,50]. Moreover, the genetic diversity pipeline used in this study was designed to remove the noise from the data. Such noise could otherwise lead to the erroneous identification of diverse positions. Such positions were identified and removed from the total set of diverse positions [45]. This may also explain the observed differences between the current analysis and the previously reported values for the *M. jansenii* population [29]. Thus, although minor discrepancies exist compared with earlier estimates, our results reaffirm the uniformly low diversity of *M. jansenii* and highlight the need for standardised, noise-aware pipelines when comparing studies.

The present study demonstrates, for the first time, a clear difference in diversity between wild and domesticated *M. integrifolia*. This finding highlights the importance of conserving wild germplasm as a source of genetic variation for future breeding programmes.

Moreover, 19 accessions showed no accession-specific, unique polymorphic homozygous SNP positions relative to the reference genome. This may reflect close genetic relatedness among these accessions; however, further validation is required. The allelic variation identified in this study provides a resource for identifying novel alleles in wild macadamia that may be absent in domesticated material and could be utilised to enhance genetic diversity in breeding programmes.

The accurate measurement of heterozygosity is crucial for understanding population structure. This study reports genome-wide or autosomal heterozygosity, which is considered more comprehensive and unbiased by sample size. Genome-wide heterozygosity estimates the proportion of heterozygous sites across all the nucleotide positions, including both polymorphic and monomorphic sites [33]. In the present study, heterozygosity in domesticated material ranged from 0.33% to 1.15%, while heterozygosity in wild *M. integrifolia*, *M. tetraphylla*, *M. ternifolia* and *M. jansenii* ranged from 0.36% to 0.71%, 0.30% to 0.71%, 0.29% to 0.42% and 0.19% to 0.34% respectively. The heterozygosity values observed in this current study fall within a similar range to those reported for walnut (0.385%) [51], hazelnut (0.334% to 0.855%) [52], and cassava (0.61–0.84%) [53]. In contrast, crops like pistachio (1.2%) [54] and mango (2.5%) [55] exhibit substantially higher heterozygosity. For macadamia, a previous study reported on heterozygosity across all four species [26], with the highest value observed in *M. tetraphylla* (He = 0.76), followed by *M. integrifolia* (He = 0.73) and *M. ternifolia* (He = 0.72), and the lowest in *M. jansenii* (He = 0.47). In contrast, the current study shows that the heterozygosity ranges of the two edible species are comparable. Additionally, the present results for *M. jansenii* were highly consistent with the previous findings reporting a low level of genome-wide heterozygosity [29].

This analysis revealed a wide range of heterozygosity in the domesticated macadamia and showed that several cultivars and breeding selections exhibited higher levels of heterozygosity than wild materials, although wild plant populations are generally reported to exhibit greater genetic heterozygosity [56]. Domestication usually reduces heterozygosity due to the loss of diversity via selection [57]. However, in macadamia, interspecific hybridization during domestication may have contributed to increased heterozygosity. Several previously studies have reported heterozygosity in cultivars and breeding selections [22,25,26,27,31,36,37,38,41]. The present study showed that both accessions of *M. integrifolia* 741 exhibited low heterozygosity values (0.40 and 0.41) compared with the previously reported value (0.98%) [38]. Furthermore, the current study identified the highest heterozygosity in Australian breeding selections (Mac_333, Mac_348, Mac_350), followed by cultivar A268 (Mac_280), which is known to be a hybrid of *M. integrifolia* and *M. tetraphylla* [41]. However, the heterozygosity value reported here for A268 is higher than the previously reported value of 0.18 based on SNP markers [41]. Similarly, the previously reported value of 0.16 for cultivar A4 was also higher in this study. According to Hardenbol et al., a possible explanation is that when using codominant markers such as SNPs and SSRs, only one of the two alleles may be detected; consequently, heterozygosity may be underestimated due to the presence of null alleles [41,58].

The relationship between individual-level heterozygosity and phenotypic traits has been examined in both plant and animal species [59,60,61,62]. Previous studies have reported no relationship between heterozygosity and kernel recovery and yield efficiency but have shown a significant relationship with cumulative kernel yield, canopy volume, cumulative nut-in-shell yield and tree height at age 6 in macadamia [25]. This study supports the previous findings of kernel recovery with no significant relationship observed with heterozygosity [25]. However, the current findings show a significant relationship with yield efficiency but no significant relationship with tree height or canopy volume. Both studies reported a significant relationship between heterozygosity and the cumulative NIS yield. The results of the present study indicate that there is an optimum level of heterozygosity associated with yield performance, above which performance may decline. Therefore, selecting highly heterozygous cultivars or breeding selections is not necessarily desirable; instead, the consideration of an appropriate level of heterozygosity may be more useful when selecting parents for crossing. Low heterozygosity may increase the risk of inbreeding depression through greater homozygosity and expression of deleterious recessive alleles. In contrast, very high heterozygosity resulting from wide crosses may disrupt favourable gene combinations and, in some cases, contribute to outbreeding depression. This suggests that maintaining an intermediate level of heterozygosity may be important for balancing genetic diversity with agronomic performance. However, this does not imply that heterozygosity itself should be used as a direct selection criterion in breeding programmes. Selection is generally based on desirable phenotypic traits. Information on heterozygosity can be used to guide the choice of parents for crossing, helping to avoid parental combinations that are either too closely related or excessively divergent. In this way, heterozygosity is the most useful as a complementary measure for selecting parental combinations that maintain sufficient genetic diversity and potentially promote favourable heterosis in the progeny.

Although the distribution of accessions suggests that phenotypic performance may vary across different levels of heterozygosity, the present analysis was based on Pearson’s correlation. Further validation using larger and independent populations, together with appropriate nonlinear statistical analyses, is required to determine whether an optimum level of heterozygosity is associated with performance and to assess its potential relevance to breeding programmes. This refers to the general genome-wide level of heterozygosity rather than heterozygosity at specific genes or loci. Agronomic performance is also influenced by favourable alleles and functional variants associated with particular traits, which require further targeted investigation.

## 5. Conclusions

This study provides a comprehensive assessment of genome-wide heterozygosity at the individual level across both wild and domesticated macadamia accessions. The high-resolution SNP dataset and identification of accession-specific variants represents a valuable genomic resource for marker discovery, parent selection, and genomic prediction in future breeding programmes. The observed relationships between heterozygosity and yield-related traits suggest that an intermediate, rather than maximal, level of heterozygosity may be associated with improved performance. Future studies using larger and independent populations are required to validate this relationship and determine its relevance to breeding. Targeted genomic analyses will also be important for identifying favourable alleles and functional variants associated with yield and other economically important traits. Overall, this study offers new insights into genome-wide genetic variation in macadamia and provides a foundation for conservation strategies and genetic improvement of in emerging global nut crop.

## Figures and Tables

**Table 1:** Summary of genome-wide sequence variation.

Population	SNP Density (SNP/kb)	Heterozygous SNPs	Homozygous SNPs	Summary
Wild *M. integrifolia*	5.39–9.88	Highest: Mac_091 Lowest: Mac_250	Highest: Mac_035 Lowest: Mac_130	Mac_035 had the highest total SNP count in the coding region.
Wild *M. tetraphylla*	3.07–9.25	Highest: Mac_236 Lowest: MacP_15	Highest: Mac_030 Lowest: MacP_15	Mac_030 had the highest total SNP count in the coding region.
Wild *M. ternifolia*	3.62–5.62	Highest: Mac_300 Lowest: Mac_126	Highest: Mac_332 Lowest: MacP_12	Mac_310 had the highest total SNP count in the coding region.
Wild *M. jansenii*	2.07–4.12	Highest: Mac_282 Lowest: Mac_216	Highest: MacP_02 Lowest: MacP_03	Mac_282 had the highest total SNP count in the coding region.
Domesticated macadamia	4.02–12.4	Highest: Mac_333 Lowest: Mac_148	Highest: Mac_347 Lowest: Mac_135	Mac_348 had the highest total SNP count in the coding region.

**Table 2:** Summary of genetic diversity across wild and domesticated macadamia populations.

Population	Total Genetic Diversity (%)	Individual Diversity Range (%)	Highest Diversity	Lowest Diversity
Wild *M. integrifolia*	4.43	0.53–0.97	Mac_035	Mac_163
Domesticated *M. integrifolia*	3.40	0.39–0.96	Mac_072	Mac_135
Wild *M. tetraphylla*	4.33	0.31–0.92	Mac_030	MacP_15
Wild *M. ternifolia*	1.80	0.36–0.55	Mac_300 Mac_310	MacP_12 Mac_126
Wild *M. jansenii*	1.10	0.20–0.40	Mac_282	MacP_16
Domesticated macadamia	6.10	0.40–1.24	Mac_348	Mac_135

**Table 3:** Summary of genetic diversity among domesticated macadamia.

Breeding Group	Sample Size	Genetic Diversity Range (%)	Total Genetic Diversity (%)
Hawaiian Agricultural Experiment Station (HAES)	37	0.39–0.96	3.33
Hidden Valley Plantation (HVP)	12	0.56–1.02	2.31
Heritage cultivars	17	0.52–1.05	3.62
Norm Greber	9	0.41–0.97	2.53
Backer	4	0.29–0.45	0.96
Ian McConachie	7	0.49–0.9	2.03
Australian breeding selections (NMBPA)	108	0.43–1.23	5.16

## Data Availability

All sequence data for wild material are available at NCBI under Bio Project PRJNA1036028 (Bio Sample numbers SAMN38356272–SAMN38356438) and domesticated material is available under Bio Project PRJNA1089341 (Bio Sample numbers SAMN40990754–SAMN40990952).

## Supplementary Materials

The following supporting information can be downloaded at: https://www.mdpi.com/article/10.3390/biology15181627/s1.

## Abbreviations

HAES	Hawaii Agricultural Experiment Station
HVP	Hidden Valley Plantations
Indels	Insertions and deletions
MNPs	Multi-nucleotide polymorphisms
NIS	Nut in shell
NMBPA	Australian macadamia breeding programme accession
NSW	New South Wales
PIC	Polymorphism information content
SNPs	Single-nucleotide polymorphisms
SSR	Simple sequence repeat
