# Supplementary material for: Comparison of Heterozygosity and Genetic Diversity Between Wild and Domesticated Macadamia
Source: Biology (Basel). 2026 Sep 15;15(18):1627. doi: 10.3390/biology15181627 (PMC13604766; doi:10.3390/biology15181627)
Supplement: Supplementary file 1 [file biology-15-01627-s001.zip › Additional file S3.pdf]

Comparison of Heterozygosity and Genetic Diversity Between Wild and Domesticated Macadamia

Sachini Lakmini Manatunga[1,2], Agnelo Furtado[1], Bruce Topp[3], Mobashwer Alam[3], Patrick J. Mason[1,2], Robert J Henry[1,2,4*]

[1]Queensland Alliance for Agriculture & Food Innovation (QAAFI), University of Queensland, St Lucia, QLD 4072, Australia.

[2]ARC Centre of Excellence for Plant Success in Nature and Agriculture, The University of Queensland, Carmody Rd, St Lucia, QLD 4072, Australia.

[3]Queensland Alliance for Agriculture & Food Innovation (QAAFI), The University of Queensland, Maroochy Research Facility, Nambour, QLD 4560, Australia.

[4]VinUni Big Data Research Institute, VinUniversity, Hanoi, Vietnam

Content

Figure no	Caption	Page no
Figure S1	The mapping titration performed at four different stringency levels of read length fraction (LF) and read similarity fractions (SF).	4
Figure S2	Frequency titration.	5
Figure S3	Diversity analysis pipeline.	6
Figure S4	Number of variants in the whole genome across wild *M. integrifolia.*	7
Figure S5	Number of variants in the whole genome across wild *M. tetraphylla.*	8
Figure S6	Number of variants in the whole genome across wild *M. ternifolia.*	9
Figure S7	Number of variants in the whole genome across wild *M. jansenii.*	10
Figure S8	Number of variants in the whole genome across domesticated samples.	11
Figure S9	Diversity within wild *M. integrifolia.*	12
Figure S10	Diversity within domesticated *M. integrifolia*.	13
Figure S11	Comparison of diversity between wild and domesticated *M. integrifolia*.	14
Figure S12	Diversity within wild *M. tetraphylla*.	15
Figure S13	Diversity within wild *M. ternifolia.*	16
Figure S14	Diversity within wild *M. jansenii.*	17
Figure S15	Diversity within domesticated cultivars and breeding selections.	18
Figure S16	Diversity within Hawaiian Agricultural Experiment Station (HAES) cultivars.	19
Figure S17	Diversity within Hidden Valley Plantation cultivars (HVP).	20
Figure S18	Diversity within Heritage cultivars.	21
Figure S19	Diversity within cultivars bred by Norm Greber.	22
Figure S20	Diversity within cultivars bred by Backer.	23
Figure S21	Diversity within cultivars bred by Malawi.	24
Figure S22	Diversity within cultivars bred by Ian McConachie.	25
Figure S23	Diversity within Australian National Macadamia Breeding Program Accessions (NMBPA).	26
Figure S24	Comparison of diversity in different breeding programs.	27
Figure S25	Diversity within genotypes grouped in clade I in nuclear phylogenetic analysis performed in our previous publication.	28
Figure S26	Diversity within genotypes grouped in clade II in nuclear phylogenetic analysis performed in our previous publication.	29
Figure S27	Comparison of diversity in clade I and clade II in nuclear phylogenetic analysis performed in our previous publication.	30
Figure S28	Distribution of heterozygosity across domesticated samples.	31

Content

Figure no	Caption	Page no
Figure S29	Coverage statistics.	32
Figure S30	Relationship between heterozygosity and phenotypic traits.	33

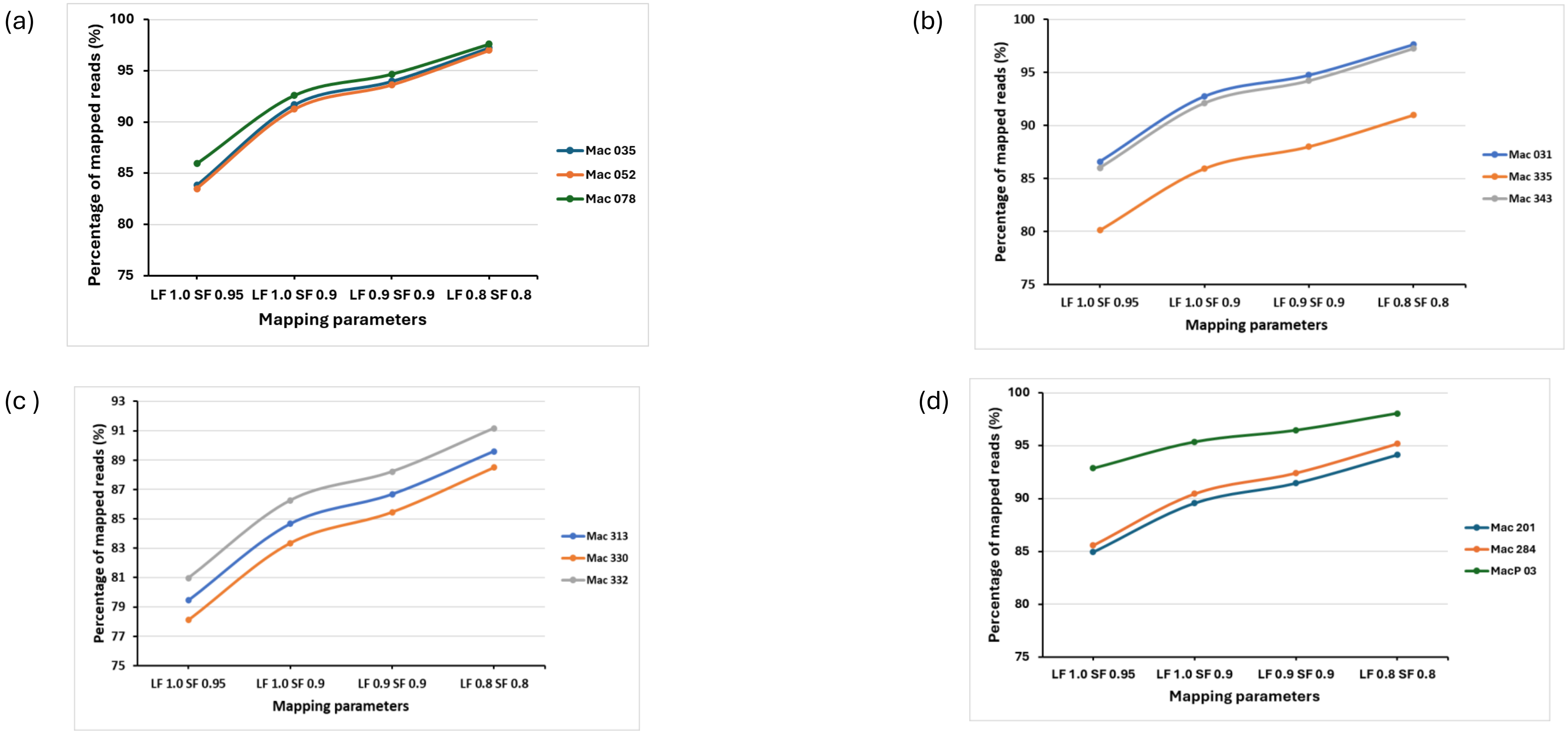

Figure S1 The mapping titration performed at four different stringency levels of read length fraction (LF) and read similarity fractions (SF) (LF 1.0 SF 0.95, LF 1.0 SF 0.9, LF 0.9 SF 0.9, LF 0.8 SF 0.8) (a) Mapping titration of three *M. integrifolia* genotypes against *M. integrifolia* reference genome. (b) Mapping titration of three *M. tetraphylla* genotypes against *M. tetraphylla* reference genome. (c) Mapping titration of three *M. ternifolia* genotypes against *M. ternifolia* reference genome. (d) Mapping titration of three *M. jansenii* genotypes against *M. jansenii* reference genome

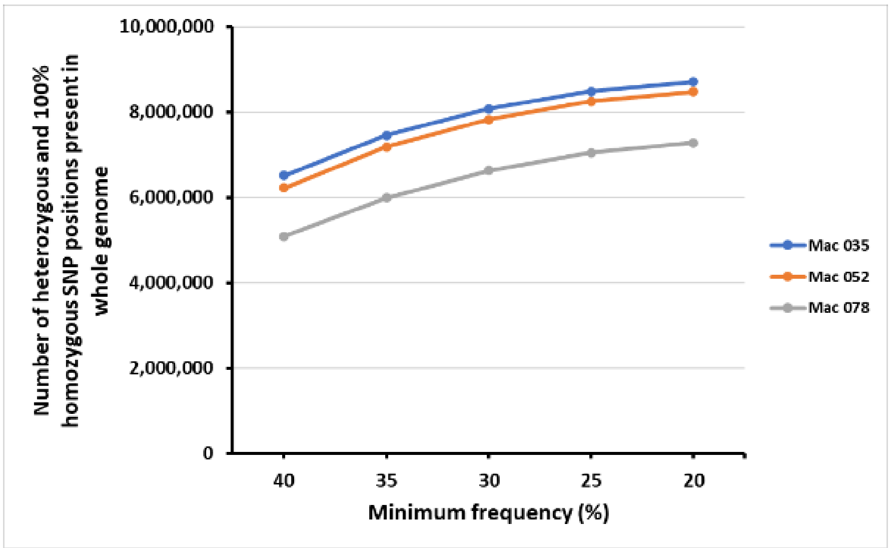

Figure S2 Frequency titration. Frequency titration was performed at five levels of minimum frequency percentage (40%, 35%, 30%, 25% and 20%) for Mac 035, Mac 052, and Mac 078.

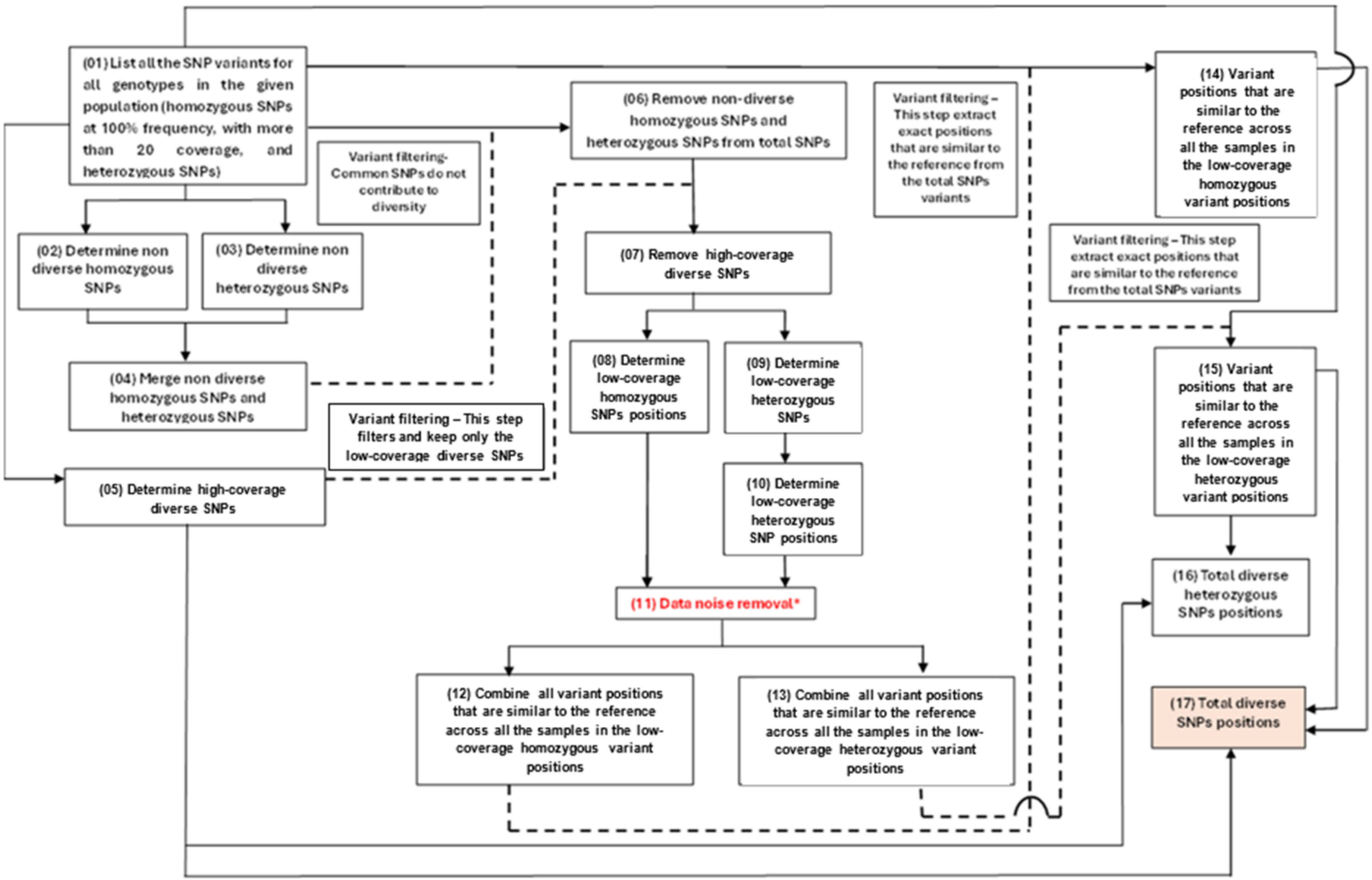

Figure S3 Diversity analysis pipeline

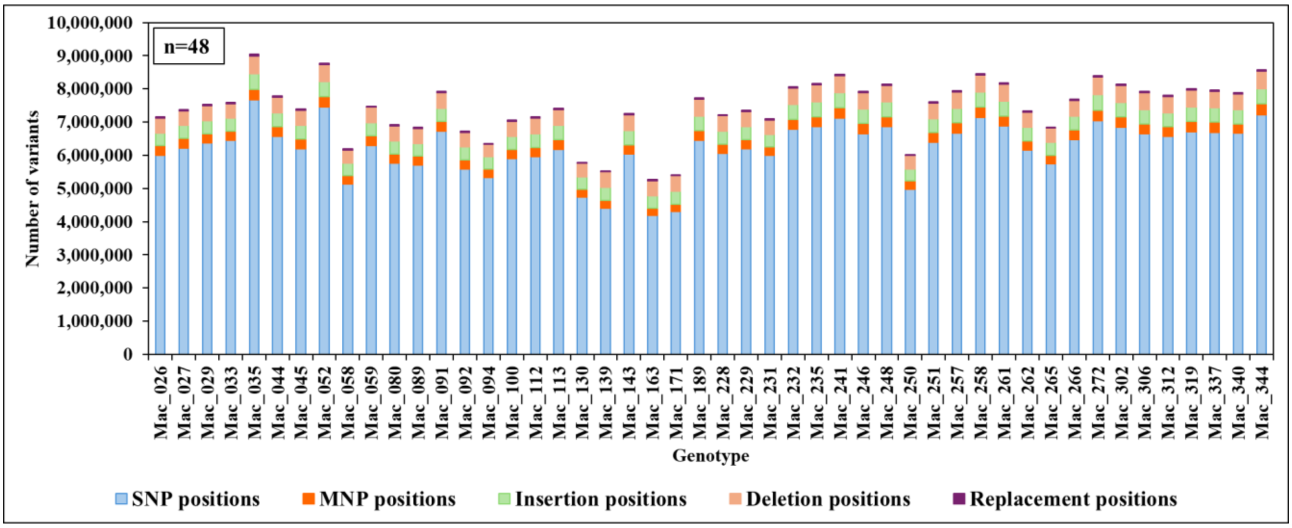

Figure S4 Number of variants in the whole genome across wild *M. integrifolia*. Colours represent different variant types: blue indicates SNP positions, orange indicates MNP positions, green indicates insertion positions, peach indicates deletion positions, and purple indicates replacement positions.

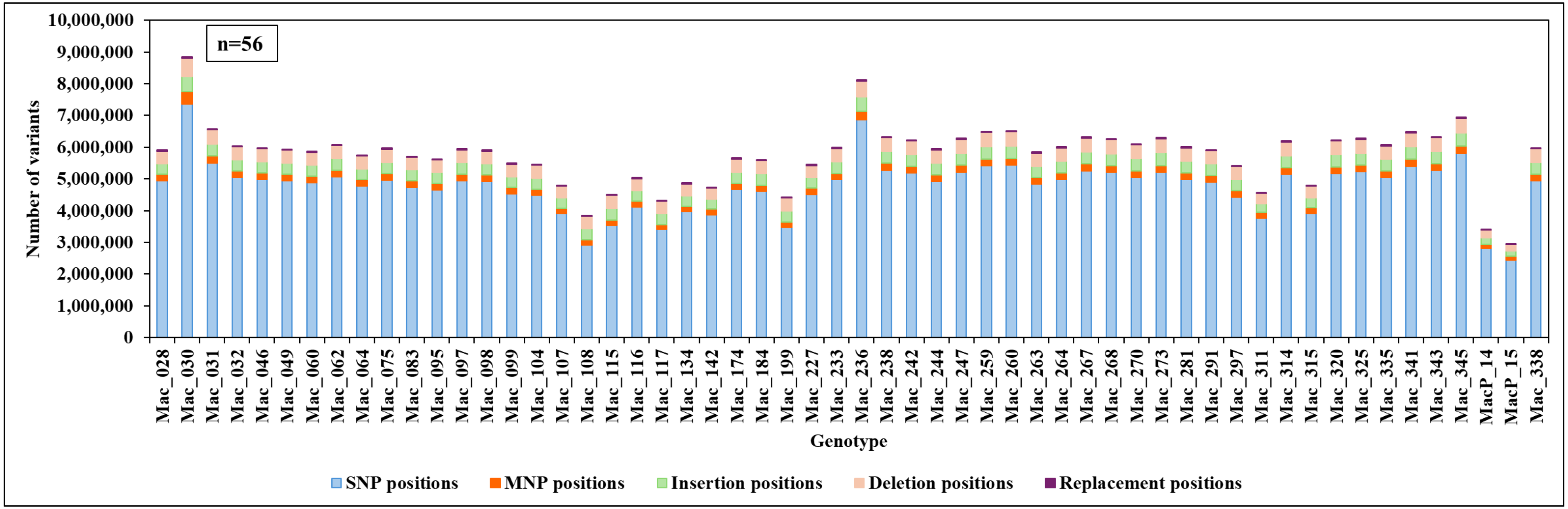

Figure S5 Number of variants in the whole genome across wild *M. tetraphylla*. Colours represent different variant types: blue indicates SNP positions, orange indicates MNP positions, green indicates insertion positions, peach indicates deletion positions, and purple indicates replacement positions.

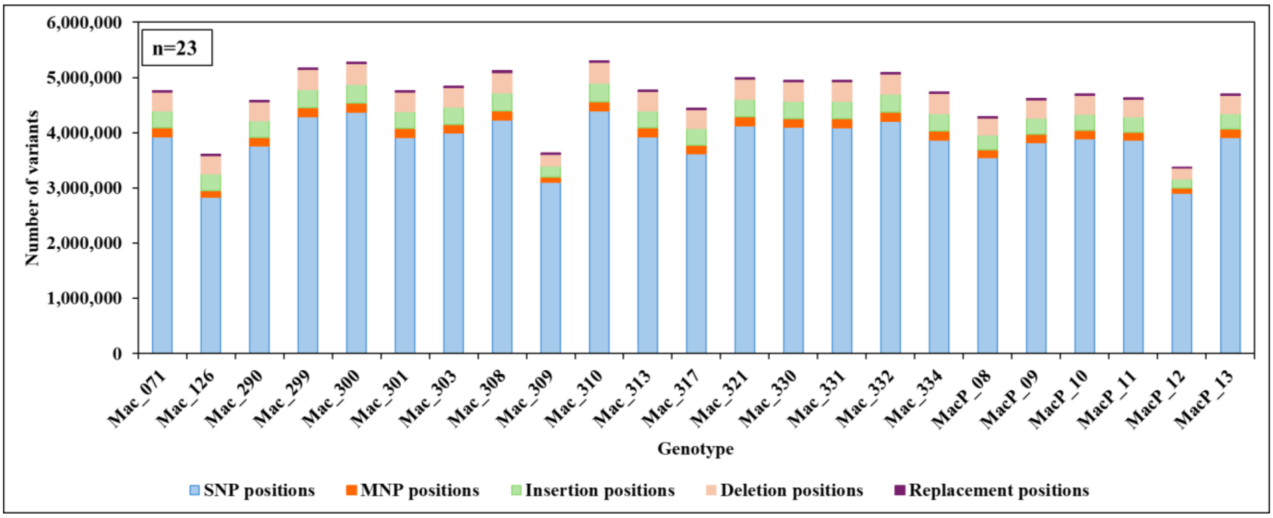

Figure S6 Number of variants in the whole genome across wild *M. ternifolia*. Colours represent different variant types: blue indicates SNP positions, orange indicates MNP positions, green indicates insertion positions, peach indicates deletion positions, and purple indicates replacement positions.

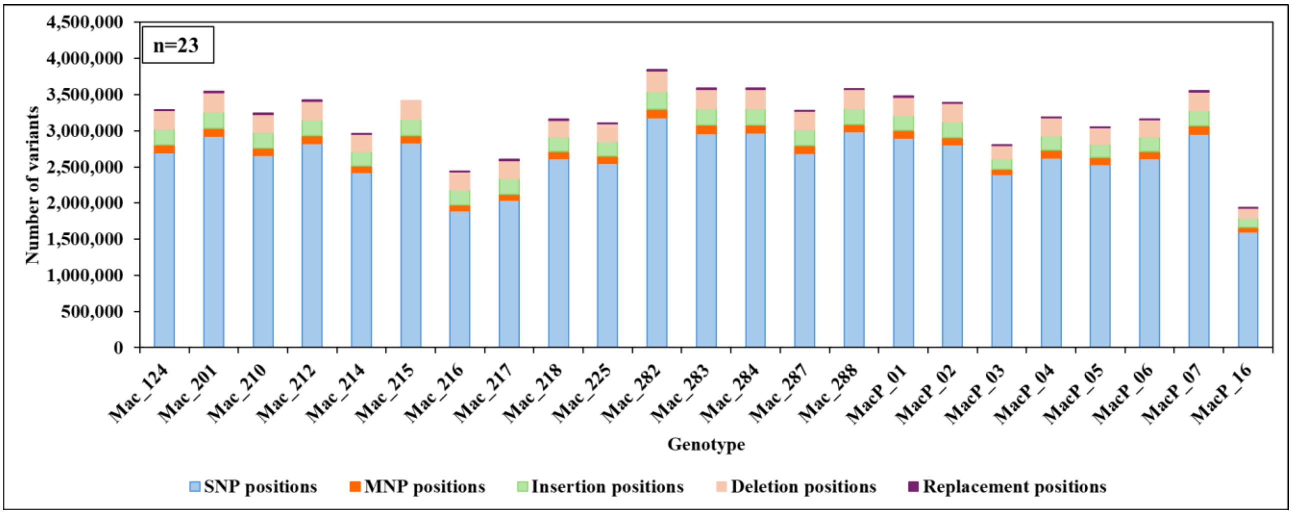

Figure S7 Number of variants in the whole genome across wild *M. jansenii*. Colours represent different variant types: blue indicates SNP positions, orange indicates MNP positions, green indicates insertion positions, peach indicates deletion positions, and purple indicates replacement positions.

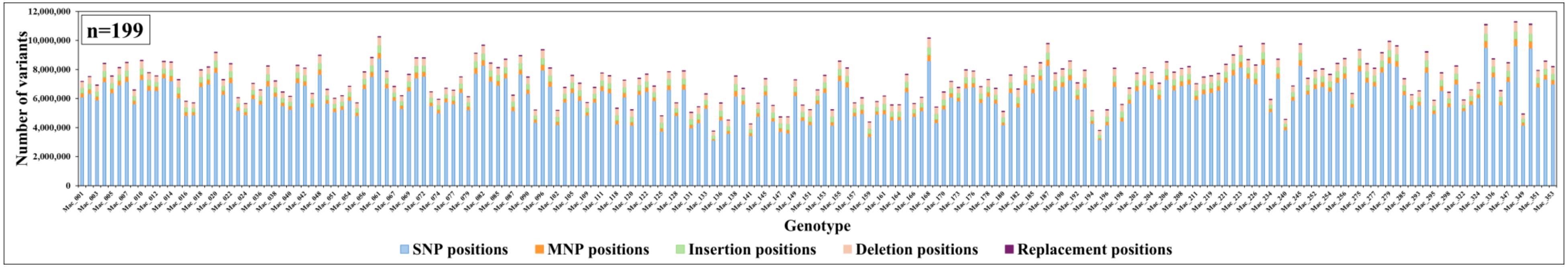

Figure S8 Number of variants in the whole genome across domesticated samples. Colours represent different variant types: blue indicates SNP positions, orange indicates MNP positions, green indicates insertion positions, peach indicates deletion positions, and purple indicates replacement positions.

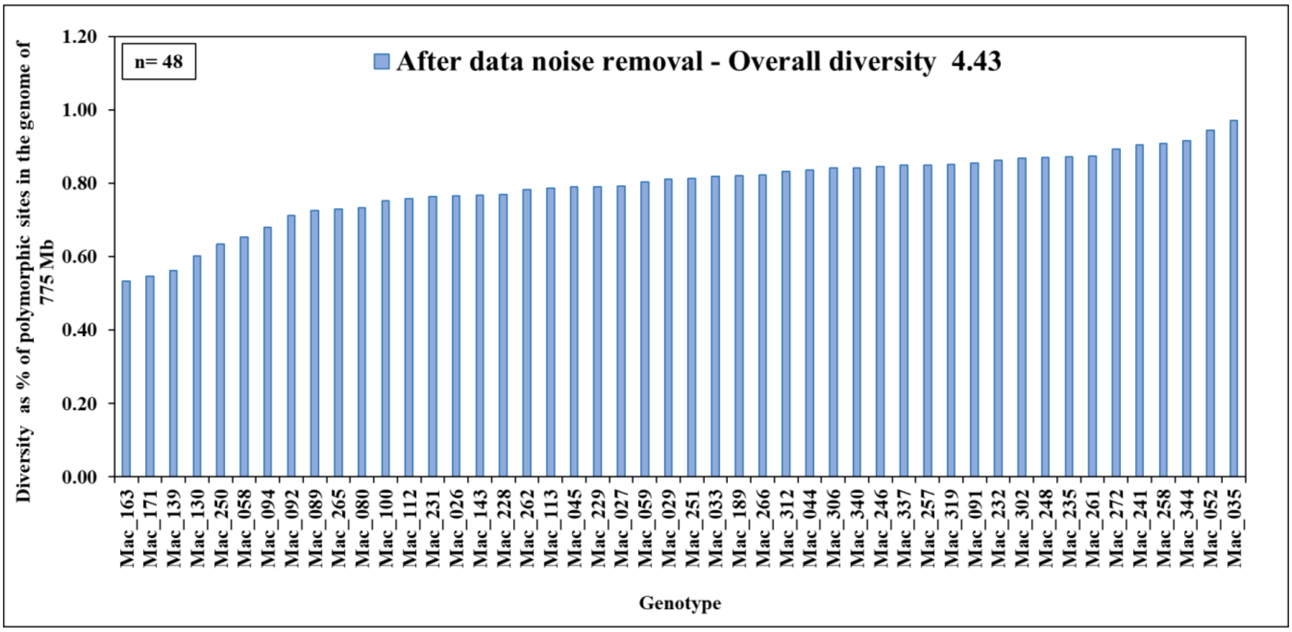

Figure S9 Diversity within wild *M. integrifolia*. Diversity was calculated as a percentage of polymorphic sites in the genome of *M. integrifolia* (775 Mb)

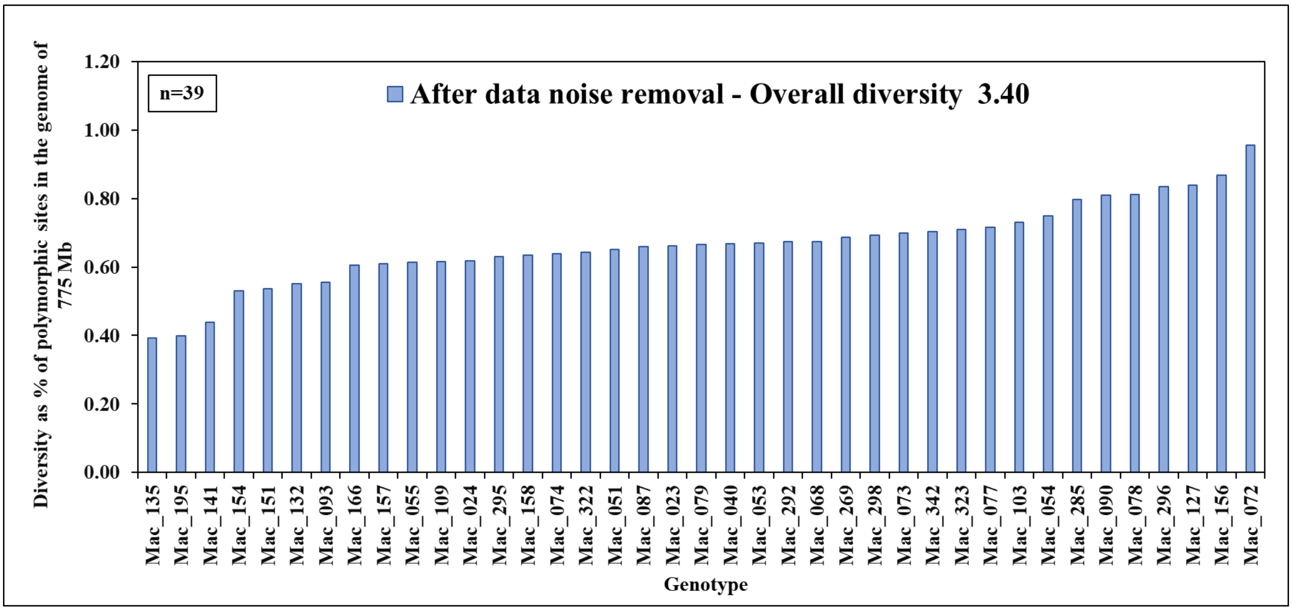

Figure S10 Diversity within domesticated *M. integrifolia*. Diversity was calculated as a percentage of polymorphic sites in the genome of *M. integrifolia* (775 Mb)

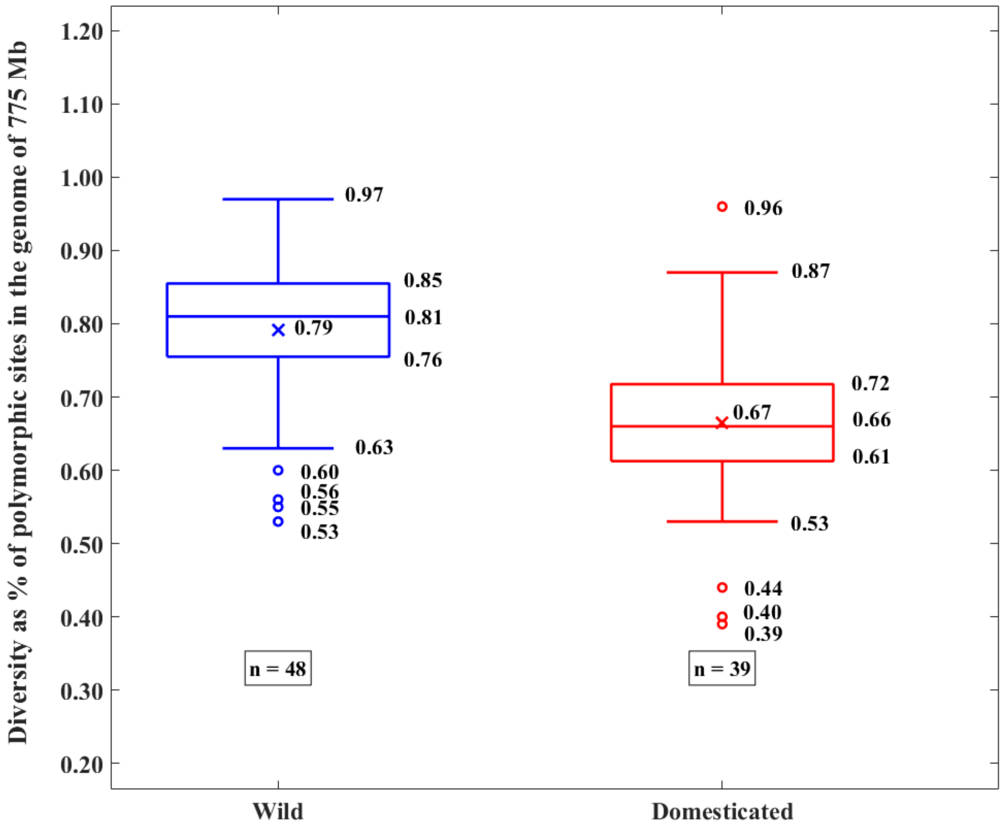

Figure S11 Comparison of diversity between wild and domesticated *M. integrifolia*. Diversity was calculated as a percentage of polymorphic sites in the genome of *M. integrifolia* (775 Mb)

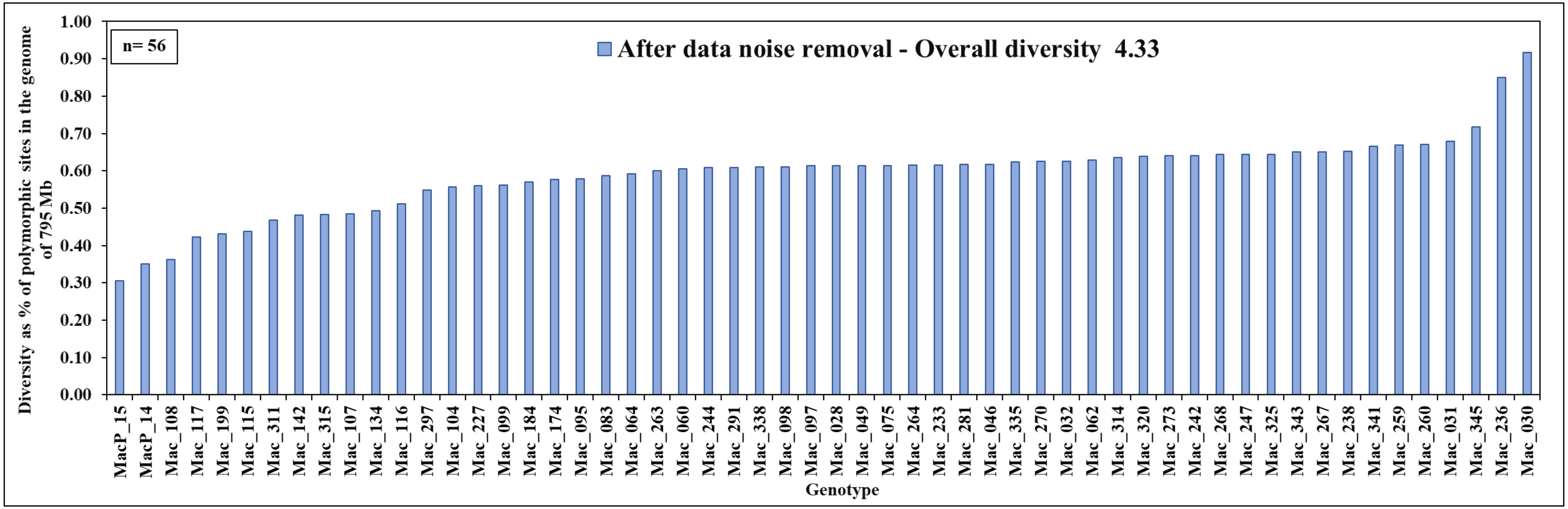

Figure S12 Diversity within wild *M. tetraphylla*. Diversity was calculated as a percentage of polymorphic sites in the genome of *M. tetraphylla* (795 Mb)

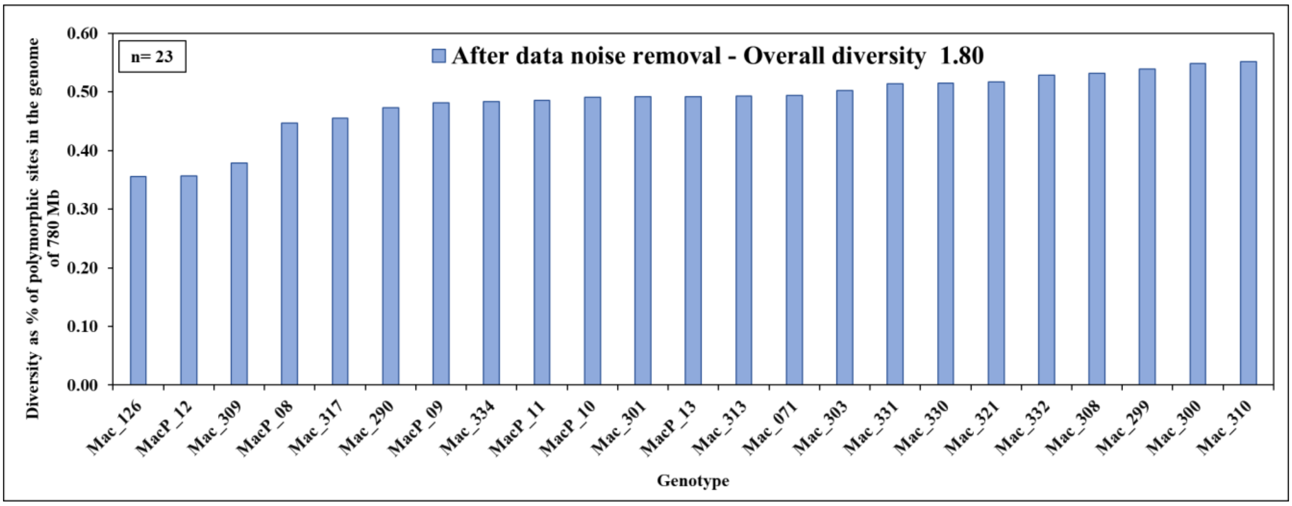

Figure S13 Diversity within wild *M. ternifolia*. Diversity was calculated as a percentage of polymorphic sites in the genome of *M. ternifolia* (780 Mb)

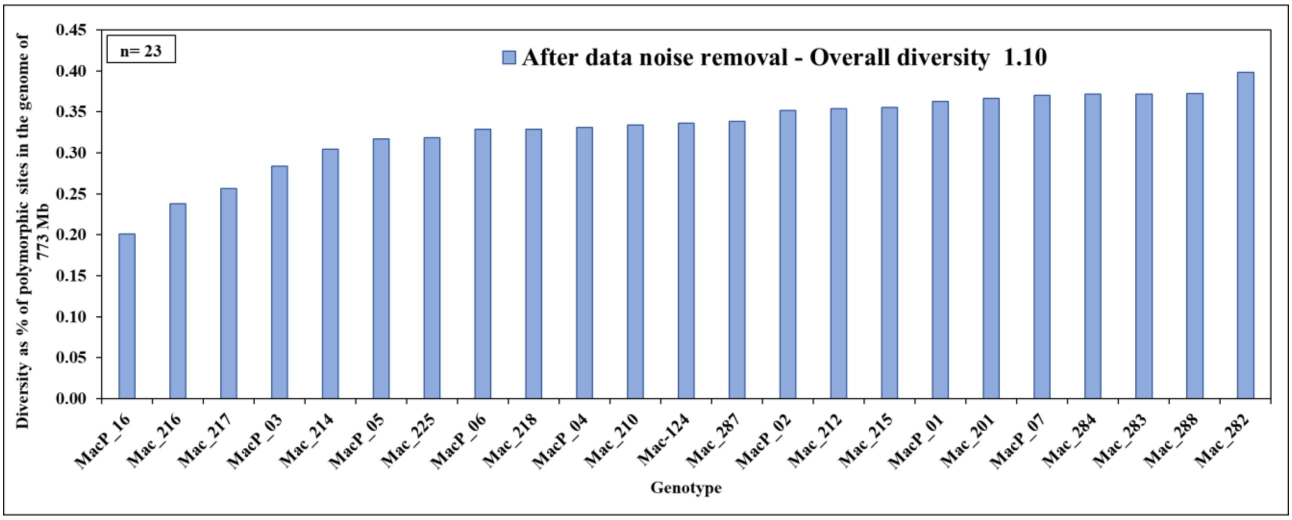

Figure S14 Diversity within wild *M. jansenii*. Diversity was calculated as a percentage of polymorphic sites in the genome of *M. jansenii* (773 Mb)

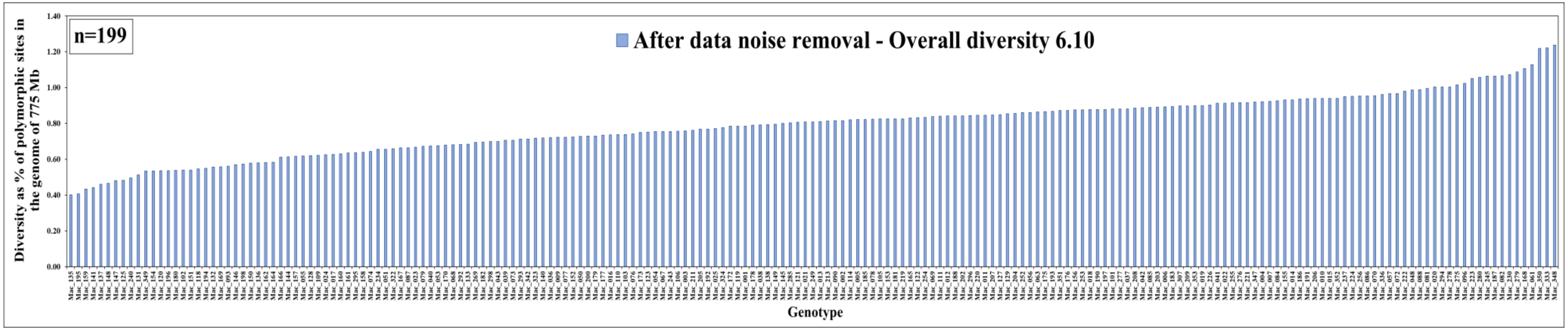

Figure S15 Diversity within domesticated cultivars and breeding selections. Diversity was calculated as a percentage of polymorphic sites in the genome of *M. integrifolia* (775 Mb)

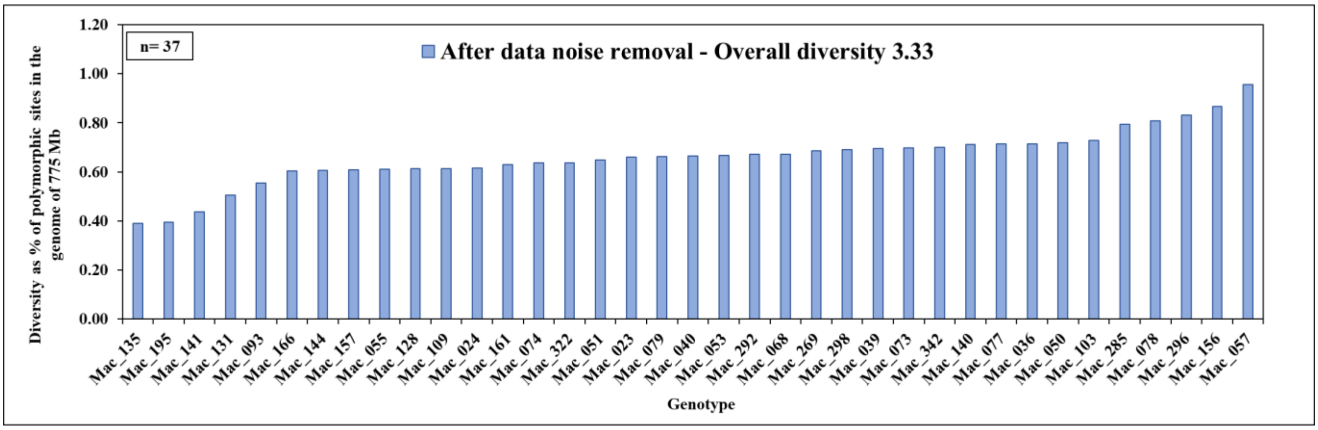

Figure S16 Diversity within Hawaiian Agricultural Experiment Station (HAES) cultivars. Diversity was calculated as a percentage of polymorphic sites in the genome of *M. integrifolia* (775 Mb)

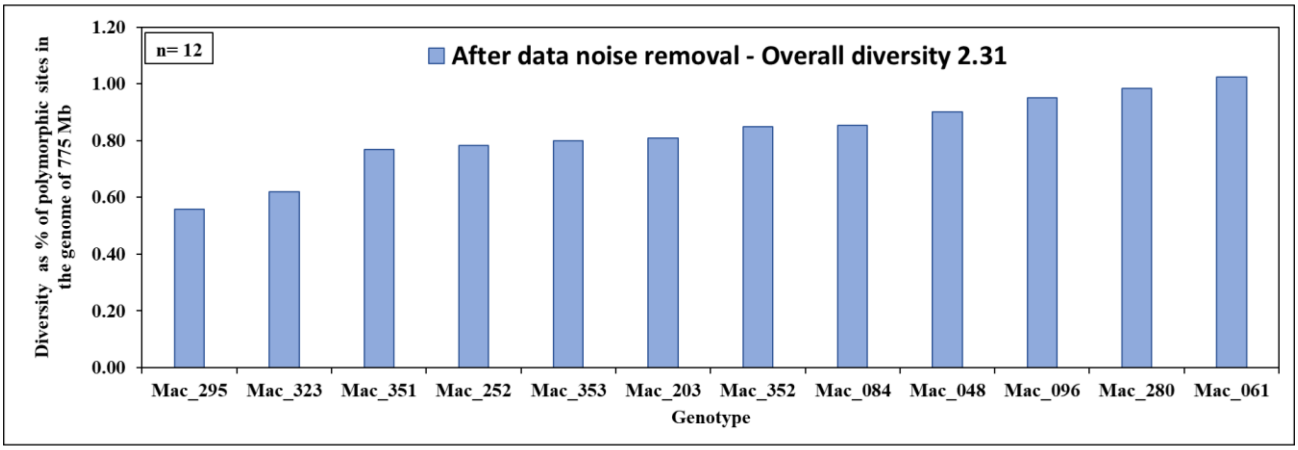

Figure S17 Diversity within Hidden Valley Plantation cultivars (HVP). Diversity was calculated as a percentage of polymorphic sites in the genome of *M. integrifolia* (775 Mb)

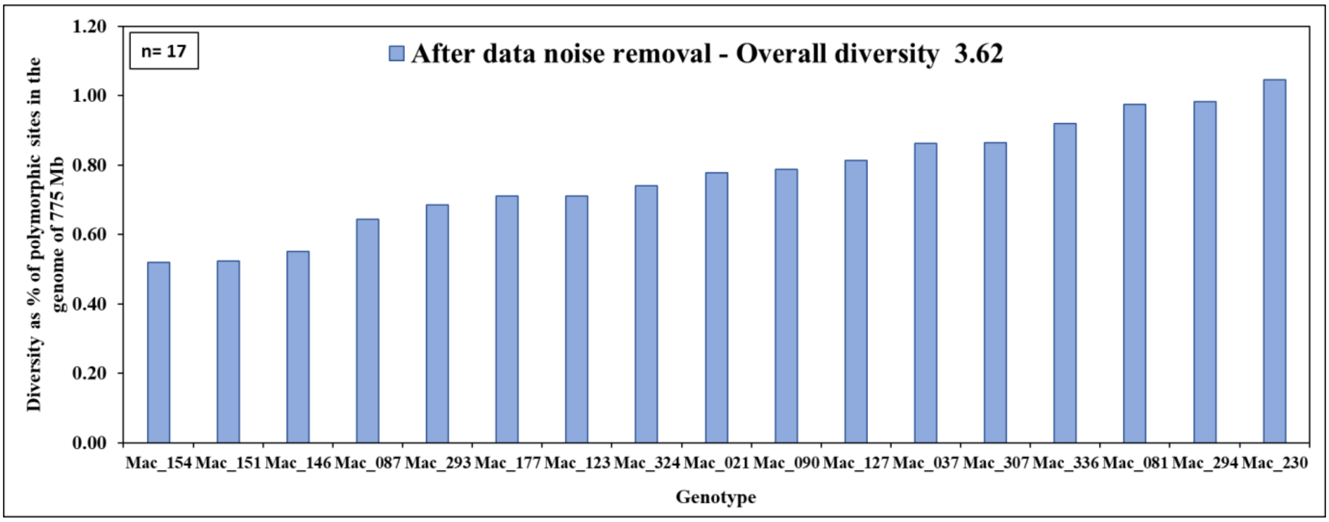

Figure S18 Diversity within Heritage cultivars. Diversity was calculated as a percentage of polymorphic sites in the genome of *M. integrifolia* (775 Mb)

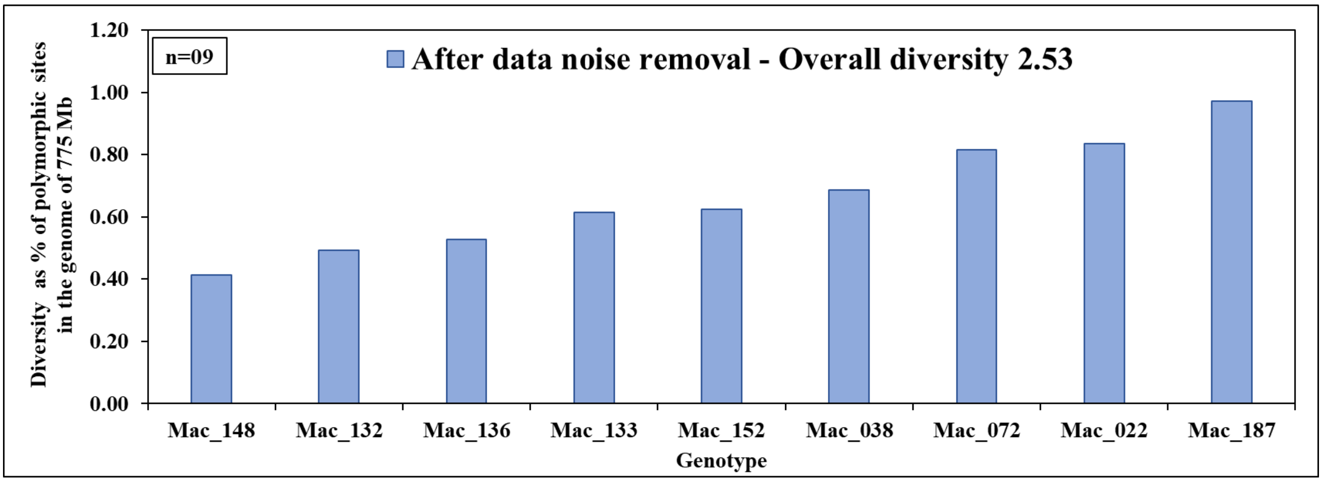

Figure S19 Diversity within cultivars bred by Norm Greber. Diversity was calculated as a percentage of polymorphic sites in the genome of *M. integrifolia* (775 Mb)

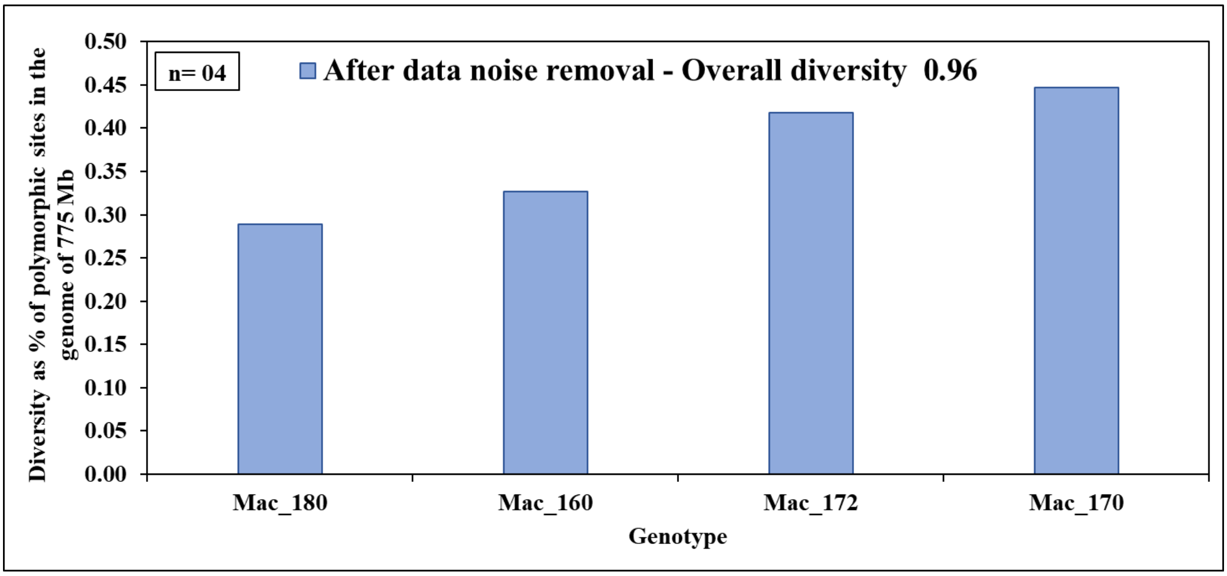

Figure S20 Diversity within cultivars bred by Backer. Diversity was calculated as a percentage of polymorphic sites in the genome of *M. integrifolia* (775 Mb)

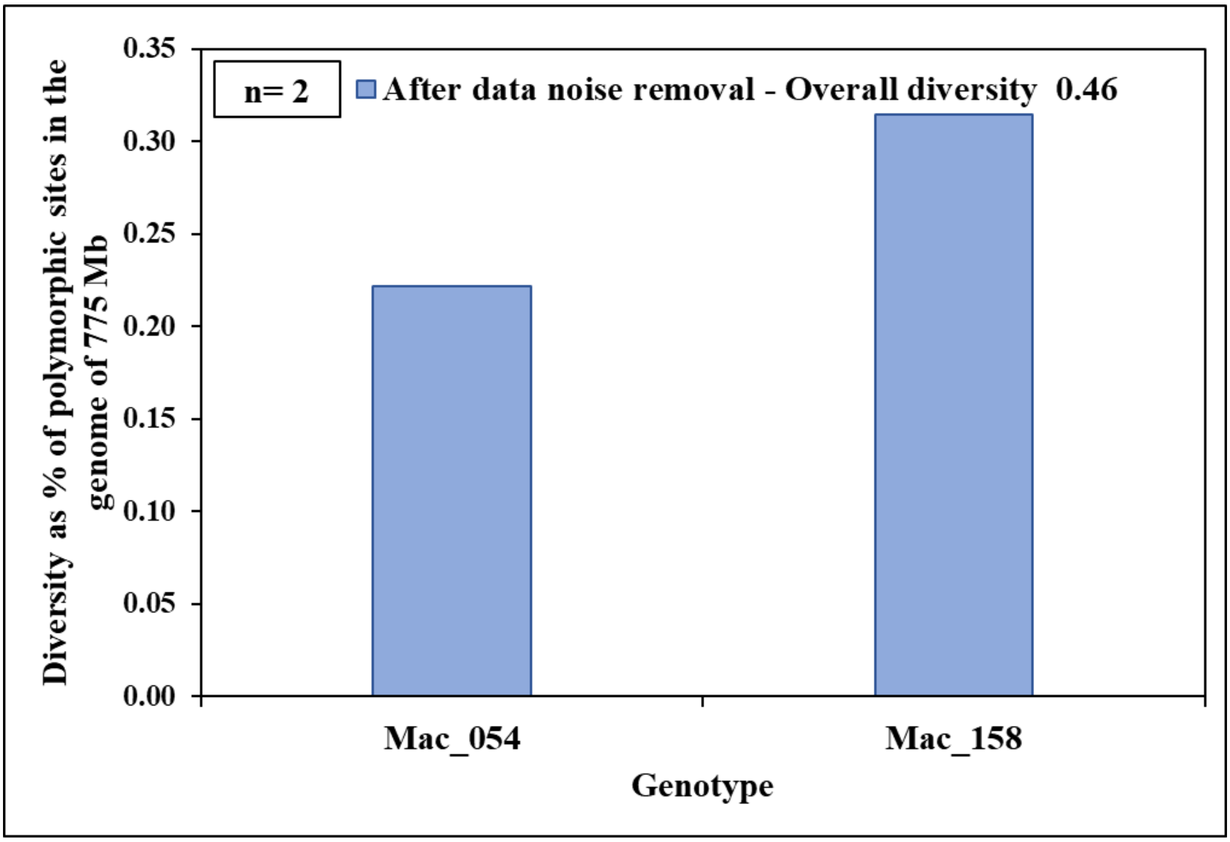

Figure S21 Diversity within cultivars bred by Malawi. Diversity was calculated as a percentage of polymorphic sites in the genome of *M. integrifolia* (775 Mb)

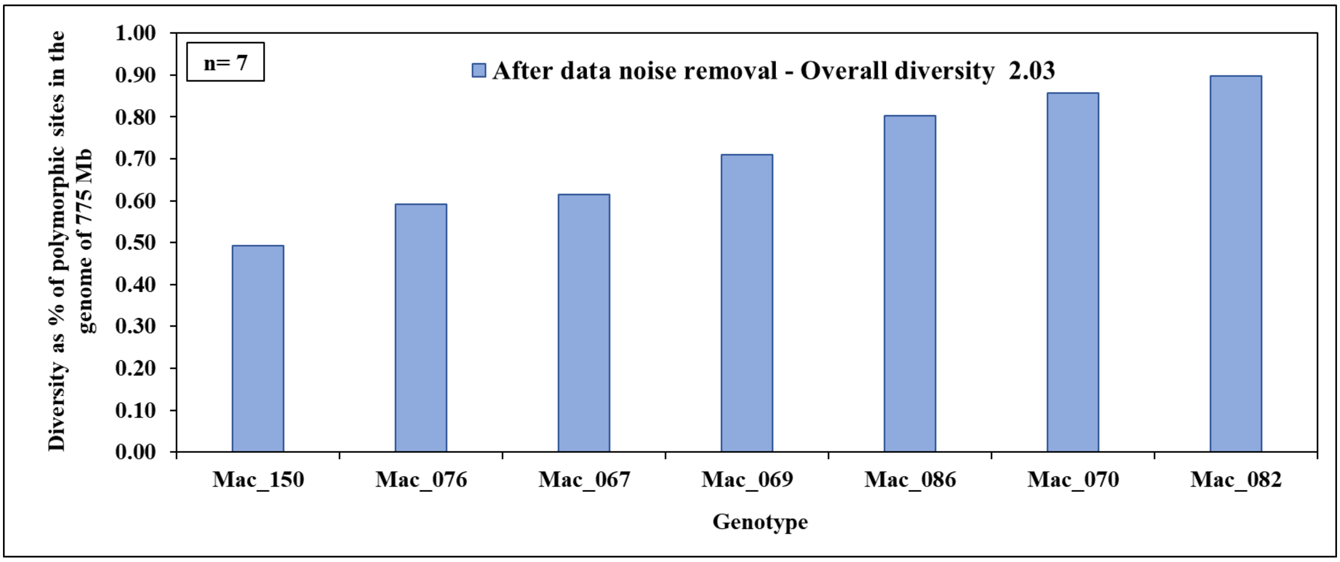

Figure S22 Diversity within cultivars bred by Ian McConachie. Diversity was calculated as a percentage of polymorphic sites in the genome of *M. integrifolia* (775 Mb)

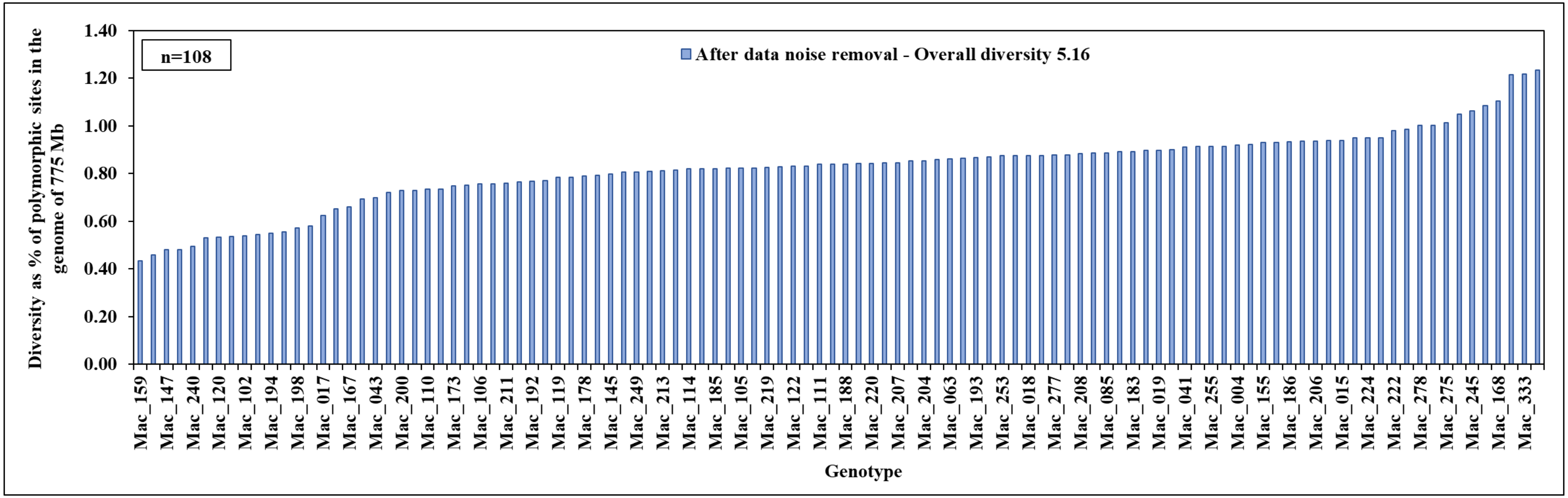

Figure S23 Diversity within Australian National Macadamia Breeding Program Accessions (NMBPA). Diversity was calculated as a percentage of polymorphic sites in the genome of *M. integrifolia* (775 Mb)

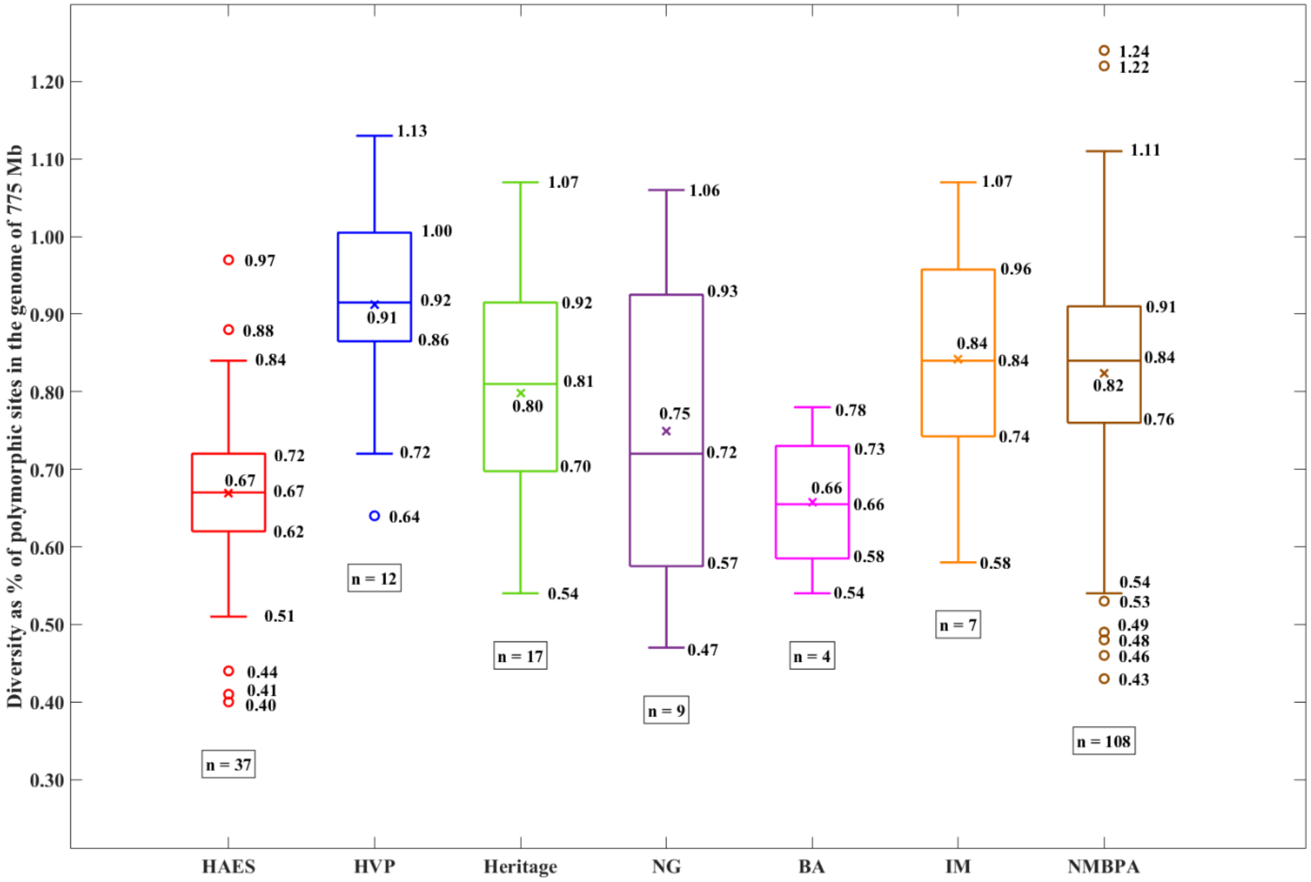

Figure S24 Comparison of diversity in different breeding programs. Diversity was calculated as a percentage of polymorphic sites in the genome of *M. integrifolia* (775 Mb). HAES: Hawaiian Agricultural Experiment Station cultivars. HVP: Hidden Valley Plantation cultivars. Heritage: Heritage cultivars. NG: Cultivars bred by Norm Greber. BA: Cultivars bred by Backer. IM: Cultivars bred by Ian McConachie. NMBPA: Australian National Macadamia Breeding Program Accessions.

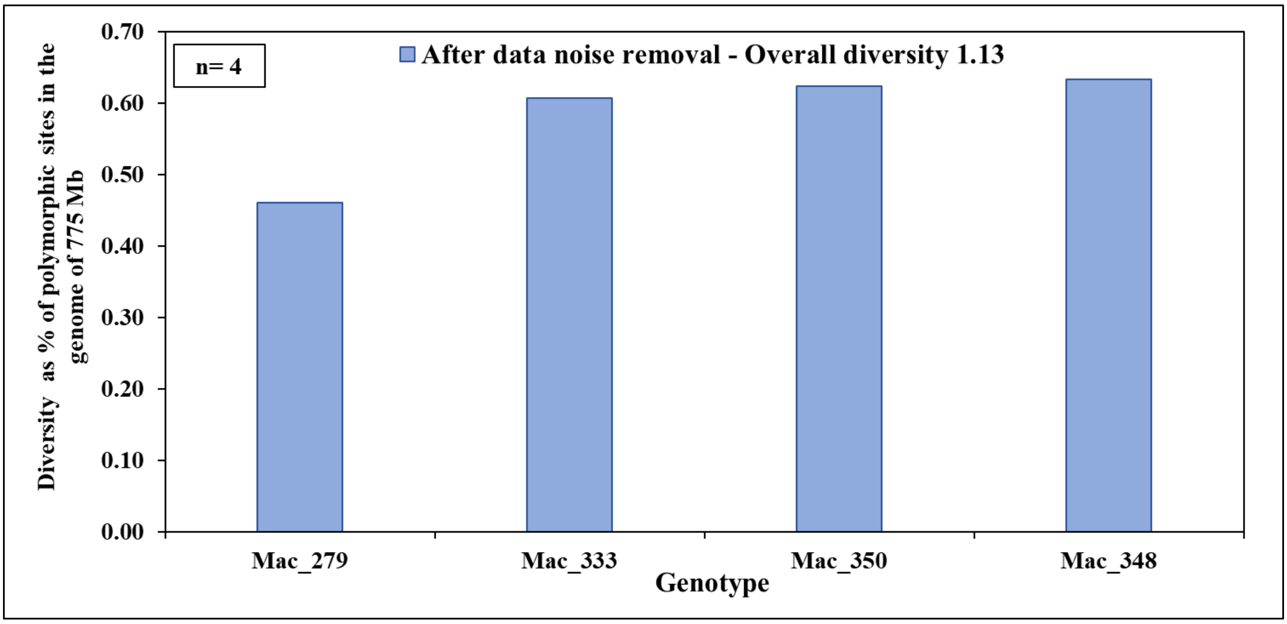

Figure S25 Diversity within genotypes grouped in clade I in nuclear phylogenetic analysis performed in our previous publication. Diversity was calculated as a percentage of polymorphic sites in the genome of *M. integrifolia* (775 Mb) (Manatunga et al., 2025)

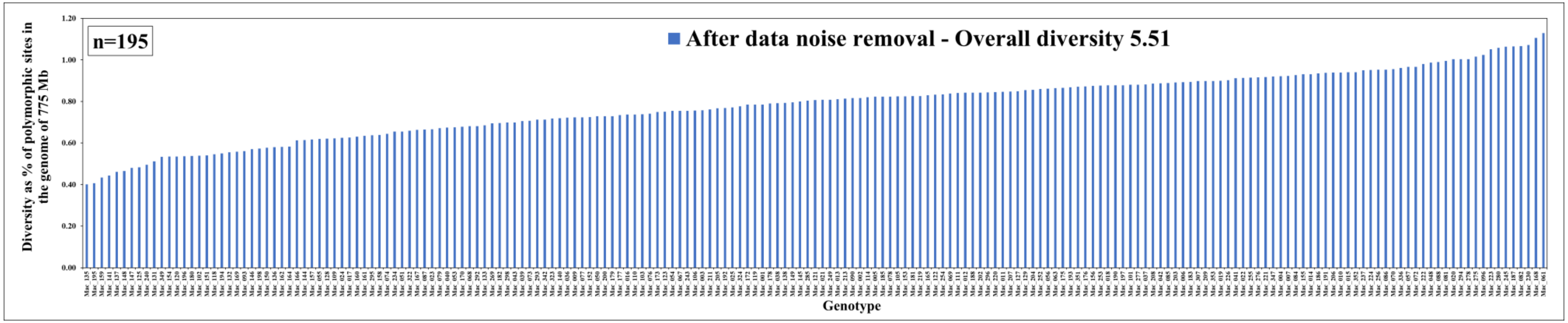

Figure S26 Diversity within genotypes grouped in clade II in nuclear phylogenetic analysis performed in our previous publication. Diversity was calculated as a percentage of polymorphic sites in the genome of *M. integrifolia* (775 Mb) (Manatunga et al., 2025)

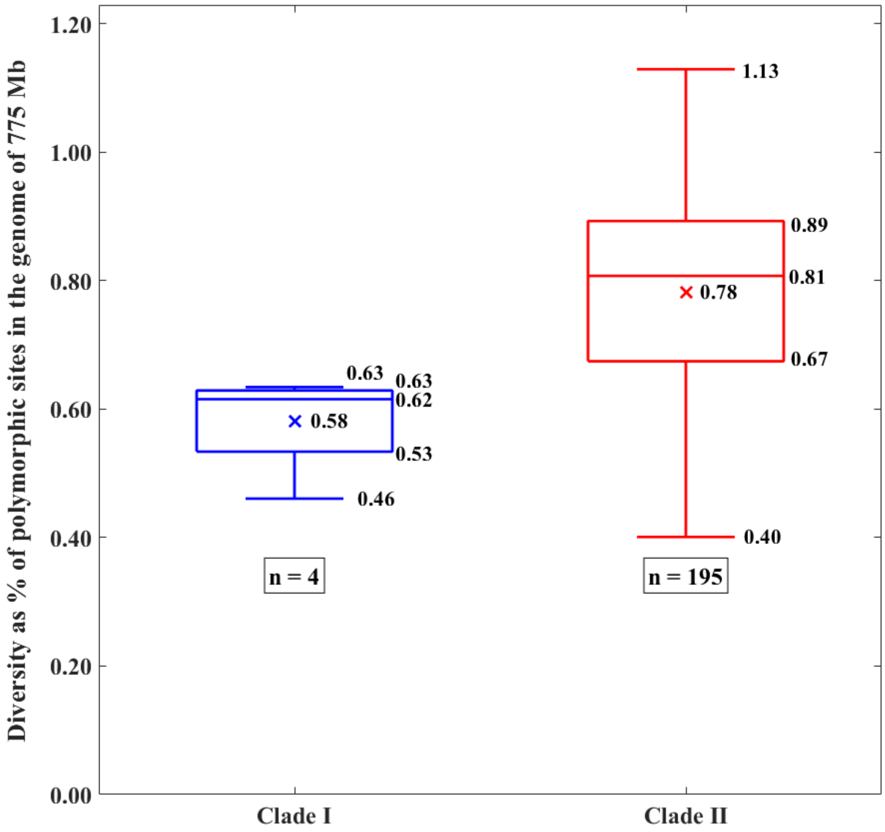

Figure S27 Comparison of diversity in clade I and clade II in nuclear phylogenetic analysis performed in our previous publication. Diversity was calculated as a percentage of polymorphic sites in the genome of *M. integrifolia* (775 Mb) (Manatunga et al., 2025)

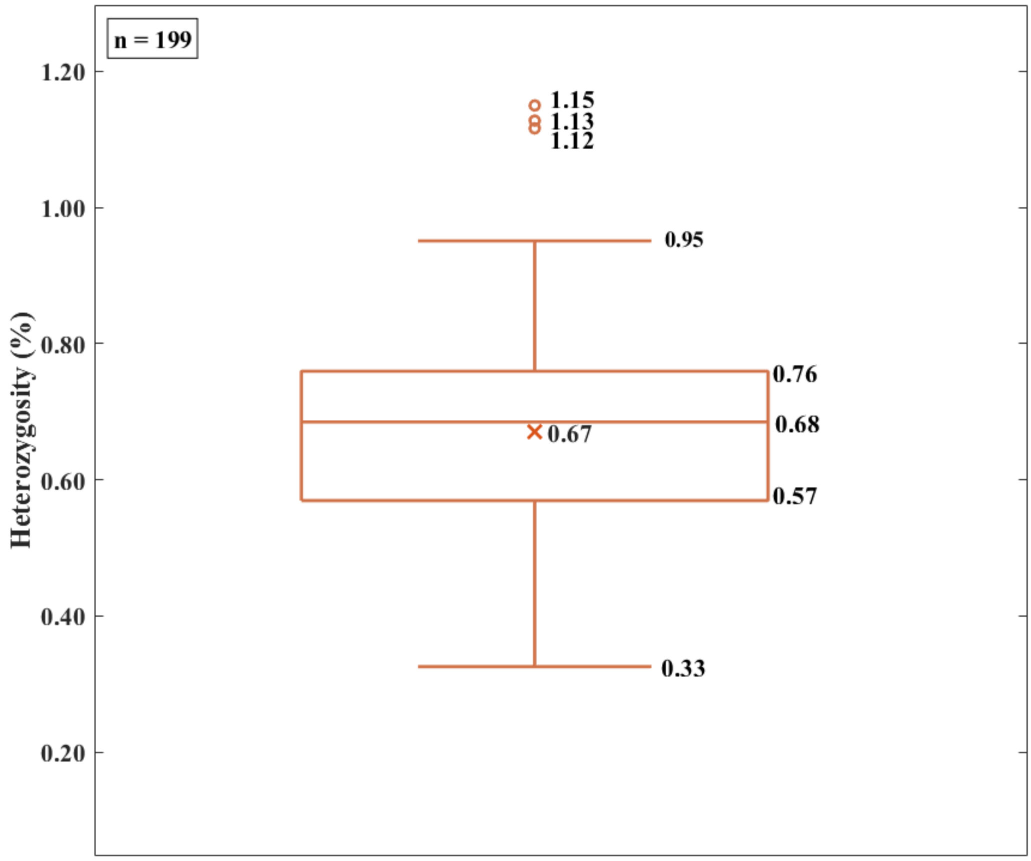

Figure S28 Distribution of heterozygosity across domesticated samples.

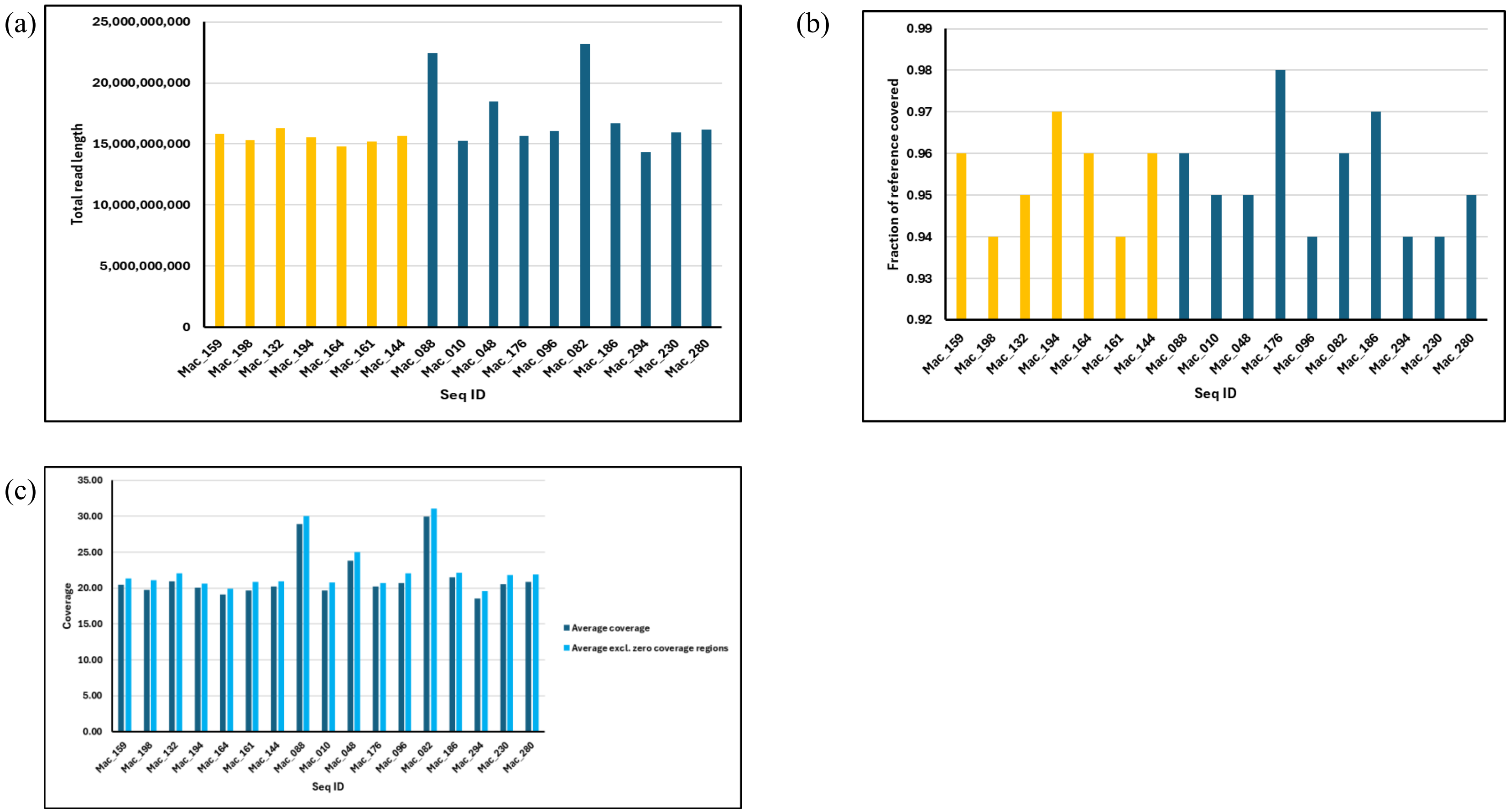

Figure S29 Coverage statistics. Mac_159, Mac_198, Mac_132, Mac_194, Mac_164, Mac_161 and Mac_144 are genotypes clustered in cluster 1 in Fig. 2 (a), (b) and (c). Mac_088, Mac_010, Mac_048, Mac_176, Mac_096, Mac_082, Mac_186, Mac_294, Mac_230 and Mac_280 are genotypes clustered in cluster 2 in Fig. 2 (a), (b) and (c). (a) Graph shows the total read length between the genotypes in cluster 1 and 2. (b) Graph shows fraction of reference covered genotypes in cluster 1 and 2. 1/1 indicates the highest coverage. In (a) and (b) yellow indicates genotypes in cluster 1 and blue indicates genotypes in cluster 2. (c) Graph shows average coverage and average coverage excluding zero coverage region. Dark blue represents average coverage, and light blue represents average coverage excluding zero coverage region.

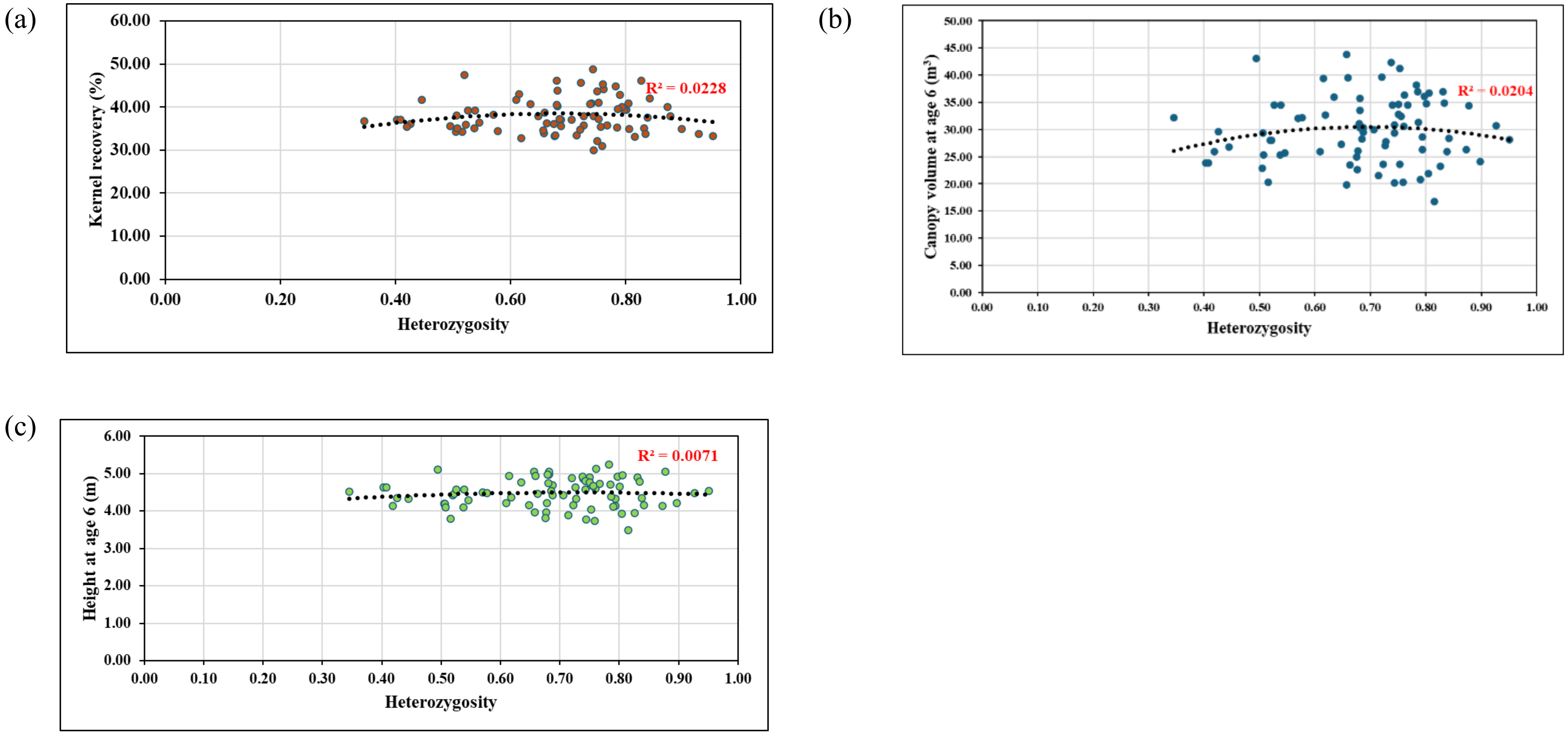

Figure S30 Relationship between heterozygosity and phenotypic traits. (a) Relationship between the heterozygosity and kernel recovery. (b) Relationship between the heterozygosity and canopy volume at age 6. (c) Relationship between the heterozygosity and tree height at age 6.
